# Electrophysiological classification of human layer 2–3 pyramidal neurons reveals subtype-specific synaptic interactions

**DOI:** 10.1038/s41593-025-02134-7

**Published:** 2025-12-10

**Authors:** Henrike Planert, Franz Xaver Mittermaier, Sabine Grosser, Pawel Fidzinski, Ulf Christoph Schneider, Helena Radbruch, Julia Onken, Martin Holtkamp, Dietmar Schmitz, Henrik Alle, Imre Vida, Jörg Rolf Paul Geiger, Yangfan Peng

**Affiliations:** 1 Institute of Neurophysiology, Charité – Universitätsmedizin Berlin, corporate member of Freie Universität Berlin and Humboldt-Universität zu Berlin, Berlin, Germany; 2 Institute for Integrative Neuroanatomy, Charité – Universitätsmedizin Berlin, corporate member of Freie Universität Berlin and Humboldt-Universität zu Berlin, Berlin, Germany; 3 Department of Neurology with Experimental Neurology, Charité – Universitätsmedizin Berlin, corporate member of Freie Universität Berlin and Humboldt-Universität zu Berlin, Berlin, Germany; 4 Department of Neurosurgery, Cantonal Hospital of Lucerne, Lucerne, Switzerland; 5 Department of Neuropathology, Charité – Universitätsmedizin Berlin, corporate member of Freie Universität Berlin and Humboldt-Universität zu Berlin, Berlin, Germany; 6 Department of Neurosurgery, Charité – Universitätsmedizin Berlin, corporate member of Freie Universität Berlin and Humboldt-Universität zu Berlin, Berlin, Germany; 7 Department of Neurology, Epilepsy-Center Berlin-Brandenburg, Charité – Universitätsmedizin Berlin, corporate member of Freie Universität Berlin and Humboldt-Universität zu Berlin, Berlin, Germany; 8 Neuroscience Research Center, Charité – Universitätsmedizin Berlin, corporate member of Freie Universität Berlin and Humboldt-Universität zu Berlin, Berlin, Germany; 9 NeuroCure Cluster of Excellence, Charité – Universitätsmedizin Berlin, corporate member of Freie Universität Berlin and Humboldt-Universität zu Berlin, Berlin, Germany; 10 Institute of Cell Biology and Neurobiology, Charité – Universitätsmedizin Berlin, corporate member of Freie Universität Berlin and Humboldt-Universität zu Berlin, Berlin, Germany; 11 Einstein Center for Neuroscience, Charité – Universitätsmedizin Berlin, corporate member of Freie Universität Berlin and Humboldt-Universität zu Berlin, Berlin, Germany; 12 Bernstein Center for Computational Neuroscience, Humboldt-Universität zu Berlin, Berlin, Germany; 13 German Center of Neurodegenerative Diseases (DZNE), Berlin, Germany

**Keywords:** Cellular neuroscience, Neural circuits, Synaptic transmission

## Abstract

Understanding the functional principles of the human brain requires deep insight into its neuronal and network physiology. In superficial layers of temporal cortex, molecular and morphological subtypes of glutamatergic excitatory pyramidal neurons have been described, but subtyping based on electrophysiological parameters has not been performed. The extent to which pyramidal neuron subtypes contribute to the specialization of physiological interactions by forming synaptic subnetworks remains unclear. Here we performed whole-cell patch-clamp recordings of more than 1,400 layer 2–3 (L2–3) pyramidal neurons and 1,400 identified monosynaptic connections in acute slices of human temporal cortex. We extract principles of neuronal and synaptic physiology along with anatomy and functional synaptic connectivity. We also show robust classification of pyramidal neurons into four electrophysiological subtypes, corroborated by differences in morphology and decipher subtype-specific synaptic interactions. Principles of microcircuit organization are found to be conserved at the individual level. Such a fine network structure suggests that the functional diversity of pyramidal neurons translates into differential computations within the L2–3 microcircuit of the human cortex.

## Abstract (web summary)

Using multineuron patch clamp in human cortex, the authors uncover four functional subtypes of pyramidal neurons with distinct morphology and subtype-specific synaptic interactions, revealing conserved microcircuit principles among individuals.

## Main

The cerebral cortex is built upon different levels of complexity: different types of neurons form local circuits that process incoming information to generate output, which is then projected through long-range connections. Canonical circuit principles within and across cortical layers have been identified, including specific connectivity and synaptic dynamics between pyramidal neurons and interneurons^1,2^. A variety of cellular subpopulations has also been described within the pyramidal neuron population^3–6^. Rodent studies have shown that layer 5 pyramidal neuron subtypes can differ in their long-range and local connectivity^7,8^. The extent to which pyramidal neuron diversity in the human cortex generates intracortical synaptic heterogeneity remains unresolved.

In humans, layer 2–3 (L2–3) is greatly expanded compared to other mammals, and this is reflected in an increased diversity of pyramidal neurons accompanied by mutations that increase corticocortical connectivity^9–13^. L2–3 pyramidal neurons have been classified based on either morphology or molecular profile^10,11,14,15^. Another identified determinant of variation in cellular properties is cortical depth^10,16,17^. However, a detailed assessment of human pyramidal neuron functional diversity in terms of electrophysiological types (e-types) is missing and would provide a phenotypic perspective on neuronal diversity complementary to previous transcriptomic classifications. Establishing the degree of neuronal as well as synaptic heterogeneity is also crucial for understanding cortical function, as it has been shown that both can be computationally advantageous^18–21^^,22^. Neuronal heterogeneity has been associated with cortical area or interindividual differences^15,23,24^. Previous studies of synaptic physiology, due to pooling of data among participants, did not dissociate interindividual differences from canonical principles of synaptic function^25–29^. To establish the generalizability of microcircuit principles, a comprehensive study of both cellular and synaptic physiologies at the individual level is necessary.

We have previously established high-throughput multineuron patch-clamp methods for the functional characterization of cortical neurons and their synapses^30^. In this study, this method allowed us to assess principles of human L2–3 neuronal, synaptic and circuit functions at the level of pyramidal neuron subtypes. To minimize variability due to different disease entities and subregion specificity^15,23^, tissue was obtained from 23 patients diagnosed with pharmaco-resistant epilepsy and who underwent anterior temporal lobe resection surgery. Recordings were focused on acute tissue from middle temporal gyrus, sampling a total of 1,479 neurons and 1,419 synapses that focus on the upper portions of L2–3. We used functional subclassification of pyramidal neuron diversity as a tool to further characterize pyramidal neuron microcircuitry and found that L2–3 pyramidal neurons were best characterized by four e-types, which formed subtype-specific wiring and synaptic dynamics.

## Results

### High-yield functional assessment of L2–3 pyramidal neuron microcircuits

**Fig. 1:**
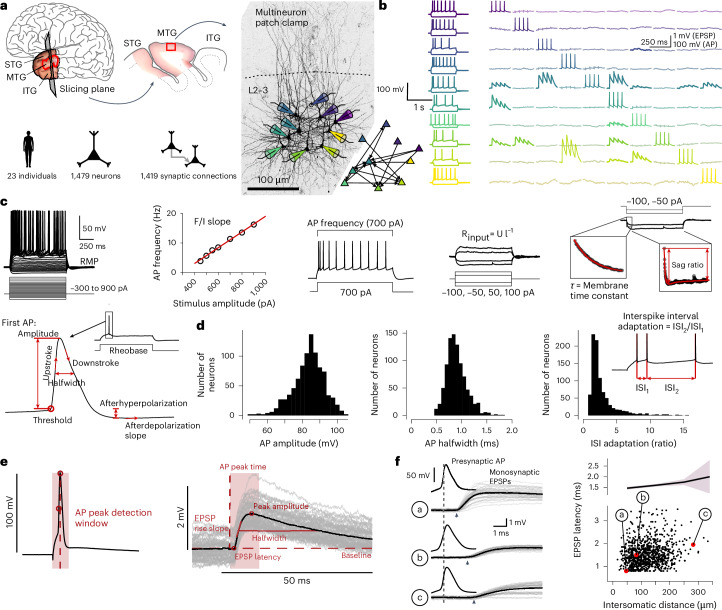
High-yield multineuron patch-clamp recordings of human cortical L2–3 pyramidal cells to extract electrophysiological signatures and synaptic properties. **a**, Experimental procedure and dataset. Human cortical brain tissue was obtained from 23 patients who underwent anterior temporal lobe resection for treatment of epilepsy. Top left: colored photograph of resected temporal lobe tissue comprising the STG, MTG, ITG. This study focused on acute slices from the MTG. Recordings were made in L2–3. Bottom: summary statistic of the dataset used in this study. Microscope image shows biocytin staining of recorded neighboring pyramidal neurons. Colored patch pipettes illustrate the multineuron patch-clamp approach (dashed circle—one neuron was not stained). Bottom right: connection pattern between the recorded neurons based on connectivity screening shown in **b**. **b**, Electrophysiological traces recorded from the ten pyramidal neurons depicted in **a**. Each row corresponds to one neuron. Left: column shows AP firing upon step depolarization. Right: matrix of traces shows averaged postsynaptic voltage traces during subsequent stimulation of APs in the neurons (diagonal). Correlated EPSPs are highlighted and indicate a monosynaptic connection. **c–e**, Schematics depicting the electrophysiological parameters extracted from the raw traces by an automated analysis pipeline. For this study, 15 cellular (**c**,**d**) as well as 5 synaptic (**e**) electrophysiological properties were analyzed (for PPR extraction, see Methods). **d**, Example cellular parameters displaying broad and partially skewed distributions. **f**, Monosynaptic connections between L2–3 pyramidal neurons analyzed in this study show a linear relationship between recording distance and synaptic latency; shaded areas correspond to 95% CI. STG, superior temporal gyrus; MTG, middle temporal gyrus; ITG, inferior temporal gyrus; CI, confidence intervals; pA, stimulus amplitude; RMP, resting membrane potential.

**Extended Data Fig. 1:**
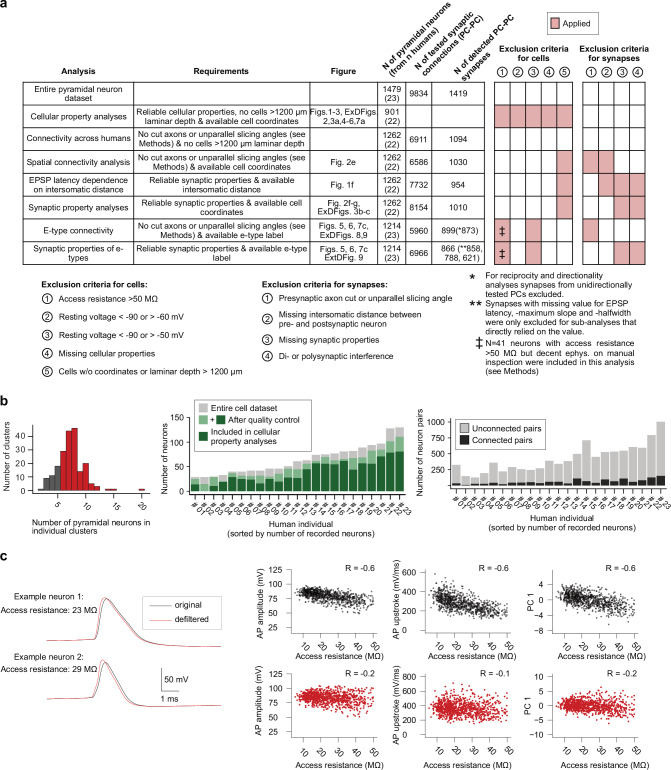
Dataset including data selection and curation. **a**, Summary table of pyramidal neuron dataset. Rows correspond to different analyses performed for this study. Each analysis, the requirements needed to perform it, as well as the figure panels of the results, are listed in columns 1 to 3, respectively. Columns 4 to 6 list the number of neurons, tested pyramidal neuron connections (PC-PC), and found synapses between pyramidal neurons that remain after exclusion criteria (shown on the right) were applied to the entire dataset. **b**, Left, histogram of cluster sizes, excluding interneurons. Clusters with size ≥6 are depicted in red. Employment of the clean-to-extend technique resulted in some clusters with size >10. Middle and right, number of recorded neurons and pairs for each sampled human individual. Individuals are sorted by the number of recorded neurons and numbering is retained throughout all figures. **c**, Defiltering of action potential (AP) traces. Left, traces of first AP elicited in response to depolarizing currents in two pyramidal neurons. The original traces (black) were defiltered using an inverted digital RC-filter (red traces; Methods). Right, AP amplitude and upstroke extracted from original traces and first principal component of all seven AP parameters extracted from original (black) and defiltered (red) traces plotted against access resistance (Pearson correlation coefficient shown, n = 901). Please note that correlation values are smaller for defiltered signals.

To study neuronal and synaptic diversities, we performed multineuron patch-clamp recordings in acute brain slices from the human temporal cortex of 23 patients undergoing resective surgery for epilepsy treatment (Fig. 1a; Methods). Our study was designed to maximize experimental yield for each tissue donor^15,16,30^. To reduce variability, we focused on middle temporal gyrus and cortical subregion L2–3, recording from neurons down to 1,200 μm cortical depth from the pia. Up to ten neurons were recorded simultaneously, allowing to functionally characterize many cells and to probe for synaptic connections between them (Fig. 1b and Extended Data Fig. 1b). Recorded neurons were filled with biocytin and were subsequently visualized (Fig. 1a). Of 1,479 pyramidal neurons recorded, 1,214 met our quality control criteria for inclusion (Methods; Extended Data Fig. 1). A total of 901 neurons from 22 individuals met further requirements and were used for cellular property analyses (Fig. 1c,d and Extended Data Fig. 1; Methods). We also tested a total of 9,834 potential connections among pyramidal neurons and detected 1,419 monosynaptic connections by evoking excitatory postsynaptic potentials (EPSPs) in response to elicited presynaptic action potentials (APs; Fig. 1e,f and Extended Data Fig. 1; Methods). On average, we obtained 53 pyramidal neurons and 62 monosynaptic connections per individual (Extended Data Fig. 1).

**Fig. 2:**
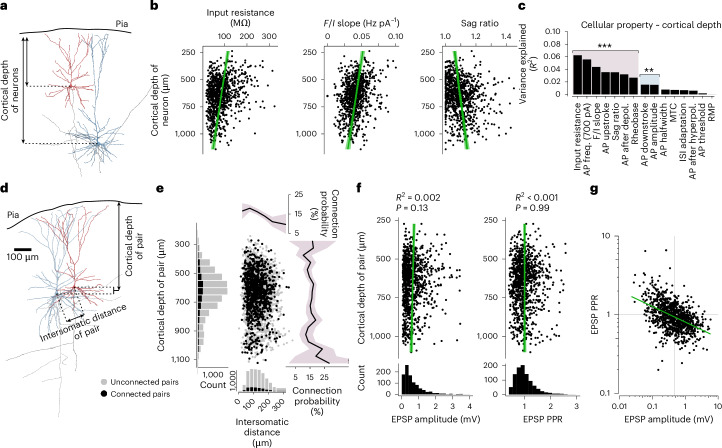
Depth dependence of cellular but not synaptic properties within L2–3. **a**, Schematic showing measurement of cortical depth of individual neurons. Median pia–soma distance across recorded neurons (*n* = 901) was 637 µm (IQR = 216 µm). **b**, Scatterplots of input resistance, *F*/*I* slope and sag ratio in relationship to cortical depth for the pooled dataset. Green lines and shaded area correspond to regression lines and 95% CI. **c**, Bar graph shows *R*^2^ values for each of the 15 cellular parameters obtained from linear regression models (two-sided *F* test, Bonferroni-corrected; adjusted *P* values are indicated by asterisks, **P < 0.01, ***P < 0.001). **d**, Schematic demonstrating measurement of cortical depth and intersomatic distance of a neuron pair. **e**, Scatterplot and histograms show the intersomatic distance and cortical depth distribution of all recorded neuron pairs. Black dots represent pairs that are connected by a synapse; gray dots represent tested but unconnected pairs. Line plots on top and on the right show distance-dependent and depth-dependent connection probabilities (line represents mean connection probability for bins; shaded area represents bootstrapped 95% CI). Mean intersomatic distance was 128 µm ± 55 and cortical depth of pairs varied between 242 and 1,122 µm. **f**, Scatterplots show EPSP amplitude and PPR in relation to cortical depth (green lines and shaded area correspond to regression lines and 95% CI). *R*^2^ values from linear regression models and *P* values according to two-sided *F* tests are shown (Bonferroni-corrected for all synaptic parameter comparisons, see Extended Data Fig. 3b). Histograms on the bottom show skewed distributions (*x* axes are cropped, see **g** for all data points). **g**, log–log plot shows power–law relationship (green line) between EPSP amplitude and PPR. The two parameters follow a power–law relationship with an exponent of −0.2 and a coefficient of 0.8. MTC, membrane time constant; IQR, interquartile range.

**Extended Data Fig. 2:**
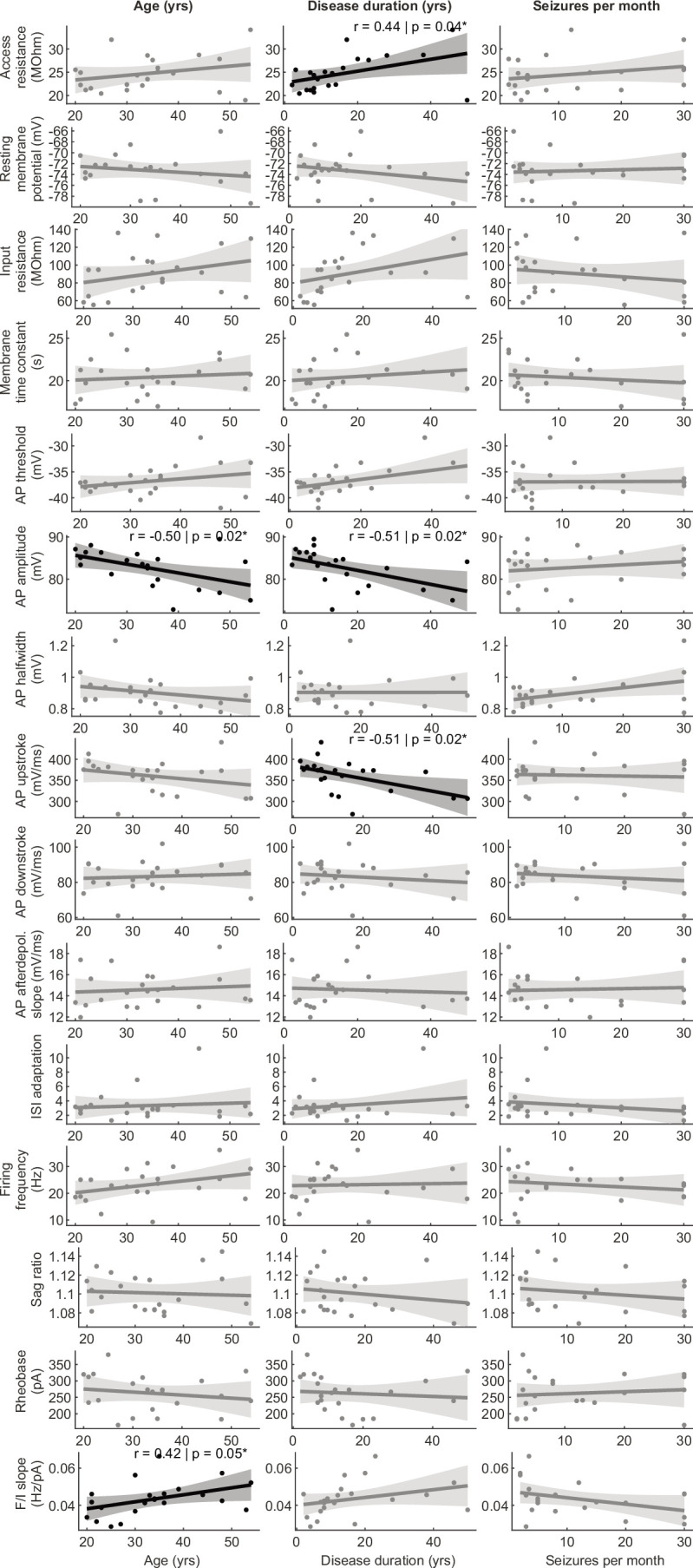
Correlation of intrinsic properties of pyramidal neurons with age and disease parameters (patient-level analysis). For the core cellular physiology dataset of n = 901 neurons, the averages of extracted parameters per patient were calculated, and Pearson’s correlations with age, as well as the clinical parameters disease duration and the number of seizures per month were determined. Regression lines (black) with 95% CI (shaded areas) are shown. Access resistance, AP amplitude, as well as slope of AP upstroke, were significantly correlated with disease duration, with p < 0.05; however, average AP amplitude was also correlated with age. An asterisk indicates that none of the correlations remained significant (that is, all p > 0.05) after Bonferroni correction for multiple comparisons.

We functionally characterized pyramidal neurons by extracting 15 passive and active electrophysiological properties that are frequently used to assess the functional phenotype of neurons^10,14,16,17^ (Fig. 1c,d; Methods). Correlating these properties with the patient’s age, the duration of epilepsy or the number of seizures per month indicated little overall influence of patient-specific parameters onto cellular properties (Extended Data Fig. 2). We further determined synaptic connectivity and extracted synaptic properties from averaged EPSP traces of monosynaptic connections, including synaptic latency, maximum slope, amplitude, halfwidth and paired pulse ratio (PPR; at 20-Hz stimulation) as a proxy for short-term plasticity (Fig. 1e; Methods). Cellular and synaptic properties displayed broad and, in some cases, skewed distributions across cells and synapses (Figs. 1d and 2f). The synaptic latency showed a positive and a nearly linear relationship with intersomatic distance (Fig. 1f; 1.7 ms mm^−1^; *F* test, *P* < 0.001), confirming the successful exclusion of disynaptic connections (Methods). In summary, we acquired an extensive dataset characterizing the diverse cellular and synaptic physiologies in the human L2–3 pyramidal neuron population.

### Structured diversity of synaptic properties without cortical depth dependence

**Extended Data Fig. 3:**
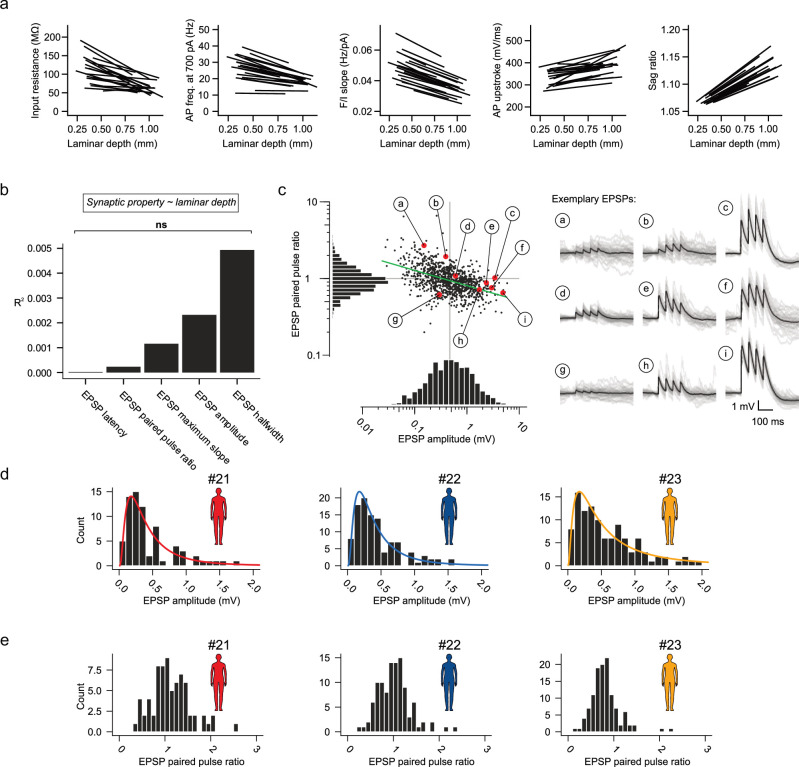
Cellular and synaptic principles of human L2-3 pyramidal neurons. **a**, Laminar depth dependence of intrinsic properties. Each plot shows, for one of five selected properties, fit lines obtained from a linear mixed-effects model with varying intercepts and slopes for each human participant (Methods). **b**, Bar graph displaying R^2^ values for each of the five synaptic parameters obtained from linear regression models (F-test, Bonferroni-corrected, all p-values > 0.05). R^2^ values from linear model fits were not significant for synaptic parameters. **c**, log-log plot shows power–law relationship (green line) between EPSP amplitude and PPR. The two parameters follow a power–law relationship with an exponent of –0.2 and a coefficient of 0.8 at 20 Hz stimulation frequency. Please note that histograms of logarithmically displayed data show symmetrical distributions. Vertical and horizontal gray lines correspond to the median EPSP amplitude and PPR of 1.0, respectively. Red dots labeled with encircled lowercase letters a-i mark data points for example EPSPs that are plotted on the right (a-i; black traces represent averages from single sweeps shown in gray). **d**, Histograms show right-skewed distribution of EPSP amplitude for three exemplary human participants. Colored lines reflect log-normal fits of the data. **e**, Histograms show distribution of EPSP PPR for three exemplary individuals.

Depth dependency within L2–3 has been reported for intrinsic neuronal as well as for synaptic properties^10,16,17,25^. In line with these studies, we found significant relationships between cortical depth and input resistance (*R*^2^ = 0.06; *F* test, *P* < 0.001, linear regression model), *F*/*I* slope (*R*^2^ = 0.04; *P* < 0.001), sag ratio (*R*^2^ = 0.04; *P* < 0.001) and additional cellular properties (Fig. 2a–c). Furthermore, we established depth-dependency within and across individual human participants for input resistance, AP frequency, *F*/*I* slope, AP upstroke and sag ratio (Extended Data Fig. 3a), confirming that cortical depth-dependent change of neuronal function is a general principle in L2–3 of human temporal cortex.

Mean connection probability among L2–3 pyramidal neurons in the temporal cortex was 15.8% (1,094 found of 6,911 tested connections; Extended Data Fig. 1), excluding slices nonparallel to the orientation of apical dendrites and neurons with cut axons (Methods). This is slightly higher than reported in previous studies^25–27,29,30^. As expected, and as examined in a separate study on this dataset^31^, the connection probability decreased with increasing intersomatic distance (Fig. 2e). However, the connection probability remained stable in relation to cortical depth.

We then asked whether synaptic amplitude and short-term plasticity would be dependent on cortical depth and found no statistically significant correlation for amplitude (*R*^2^ = 0.002; *F* test, *P* = 0.13; Fig. 2f) or PPR (*R*^2^ < 0.001; *F* test, *P* = 0.99; Extended Data Fig. 3b). Furthermore, *R*^2^ values from linear regression models were below 0.005 and not statistically significant for EPSP rise slope, latency and halfwidth (Extended Data Fig. 3b). The median EPSP amplitude of 0.46 mV in our study was within the range of previous human studies^25–27,29,30,32^. However, many EPSP amplitudes were smaller than 1 mV, and only very few are larger than 3 mV (Fig. 2f). These large-amplitude synapses may underlie the co-activation of groups of neurons that are thought to be fundamental to computations of the brain^33,34^. Like EPSP amplitude, PPR showed a skewed distribution with a median of 0.93 (Fig. 2f), in line with the previous short-term plasticity measures at 20-Hz stimulation^25,32^. Both synaptic amplitude and PPR showed a log-normal distribution with an inverse power–law relationship to each other (Fig. 2g and Extended Data Fig. 3c). Furthermore, skewed distributions of amplitudes as well as a broad range of short-term plasticity at synapses were ubiquitous features found across individuals (Extended Data Fig. 3d,e). Taken together, synaptic properties exhibit large diversity within human individuals and do not change along the cortical depth within L2–3.

### Functional subtypes of human L2–3 pyramidal neurons

**Fig. 3:**
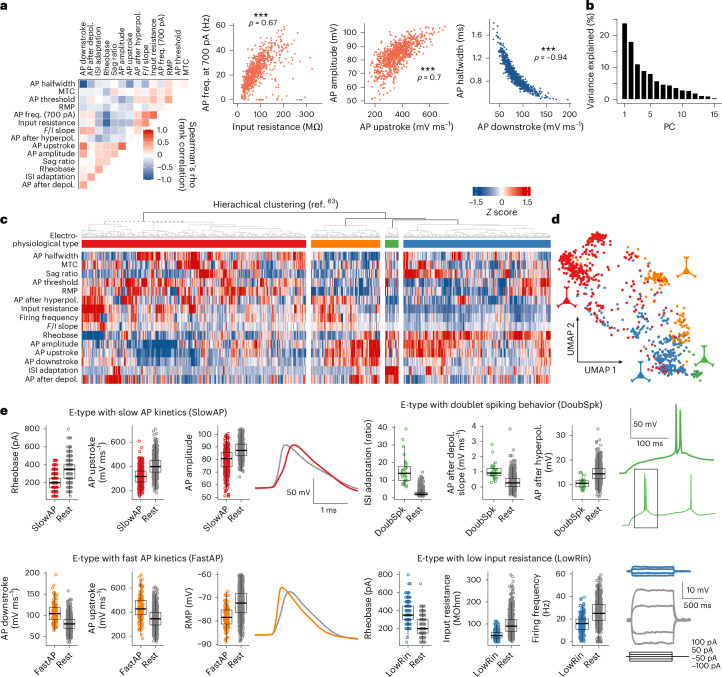
Clustering of human L2–3 pyramidal neurons into four e-types. **a**, Left: matrix shows correlation coefficients for each pair of parameters. Right: parameter pairs with strong correlation are shown as scatterplots with the color of data points corresponding to the correlation coefficient of the respective parameter pair (Spearman rank correlation test, two-sided; *P* values are indicated by asterisks, ***P < 0.001). **b**, Scree plot of a PCA of the 15 electrophysiological properties. **c**, Clustergram visualization of the cellular electrophysiological parameters for all pyramidal neurons. Each column represents one neuron, and the color code represents the *z* score of each parameter (rows). Neurons are grouped into four e-types according to hierarchical clustering using Euclidean distance and Ward’s linkage criterion. The appearance of the dendrogram on top indicates the result of statistical testing of the bifurcations according to a Monte Carlo-based approach^63^—black indicates significant bifurcations (*P* < 0.05, corrected for family-wise error), dashed line indicates nonsignificant bifurcation, gray indicates untested bifurcations due to cluster size below threshold. **d**, UMAP visualization of the high-dimensional parameter space. Each dot represents one neuron color coded by e-type. **e**, The three most distinguishing parameters of each e-type are shown. Box plots show, for all neurons included in the cellular property analysis (*n* = 901 from 22 patients; Methods; Extended Data Fig. 1), how each e-type (color coded; *n* = 293 LowRin, 27 DoubSpk, 136 FastAP, 445 SlowAP) compares with all other neurons (gray) for each parameter. Medians are shown as centerlines, boxes indicate the IQR, whiskers extend to values within 1.5 × IQR and outliers are plotted individually. Example traces are shown—colored trace represents example neuron of the respective e-type, gray trace represents a neuron of another e-type.

Distance to pia had a substantial influence on some cellular parameters; however, the amount of explained variance was limited (Fig. 2b,c). Diversity of human pyramidal neurons in L2–3, including several subtypes, has been proposed based on transcriptomic profile^10,11^ and morphology^14^. Here we sought to determine to what extent pyramidal neuron subtypes can be classified at the functional level. Because of significant correlations across cellular properties (Fig. 3a), we performed a principal component analysis (PCA) to quantify the overall dimensionality of the electrophysiological parameter space (Methods). Seven of 15 principal components were necessary to capture ~80% of the variance (Fig. 3b), suggesting that the functional parameter space of pyramidal neurons is rather high dimensional.

**Extended Data Fig. 4:**
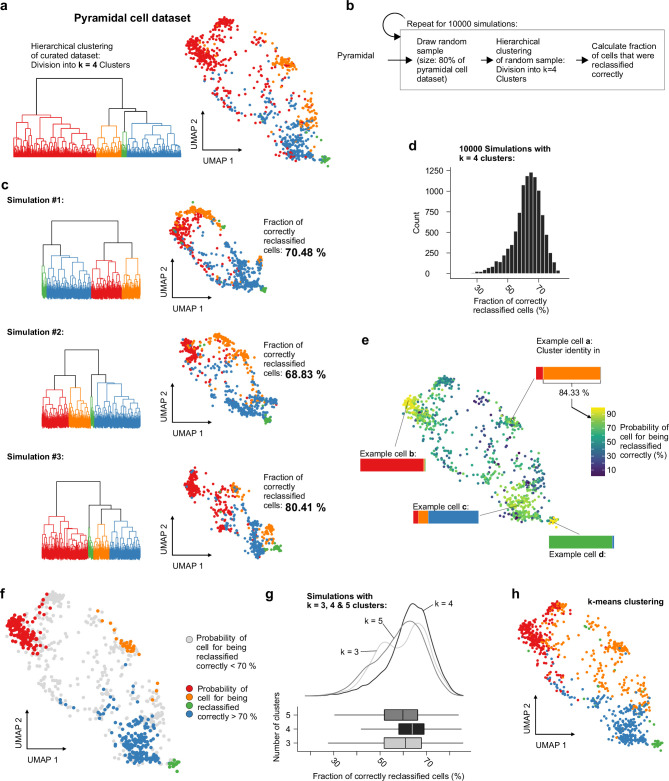
Resampling analysis of hierarchical clustering. **a**, Dendrogram and UMAP plot show the result of unsupervised hierarchical clustering (Euclidean distance, Ward’s linkage criterion) of the entire curated pyramidal neuron dataset into four clusters. **b**, Flow chart depicting the resampling procedure (Methods). **c**, Dendrograms and UMAP plots show clustering results of three exemplary simulations with randomly selected 80% subsamples that were clustered into four groups (the fraction of cells that were classified into the same cluster as in the initial clustering shown in **a** is displayed to the right of the plots). **d**, Histogram shows the number of correctly reclassified cells in 10,000 simulations. **e**, UMAP plot color coded by the probability of a cell being reclassified correctly in 10,000 simulations. **f**, Same as **e**, but only cells with a probability >70% are shown in color. The color corresponds to the cluster that a cell was assigned to in the initial clustering shown in **a**. **g**, Smoothed distributions and box plots show the number of correctly reclassified neurons in 10,000 simulations for k = 3, 4 and 5 clusters. Please note that simulations in which subsamples were clustered into k = 4 groups resulted in a higher median fraction of correctly reclassified cells. **h**, Clustering result for the entire pyramidal neuron dataset using a k-means clustering algorithm with randomly selected starting points and k = 4 clusters (Methods). Please note that the result of the k-means clustering is very similar to the hierarchical clustering shown in **a**.

We then investigated whether individual neurons are grouped into distinct subspaces of intrinsic properties, possibly reflecting functional cell types^35,36^. To address this, we performed an unsupervised hierarchical cluster analysis based on the 15 electrophysiological properties (Methods). The cluster algorithm was followed by a Monte Carlo-based approach to test the statistical significance of cluster separation, revealing statistically significant bifurcations among four clusters (Fig. 3c). To visualize the e-type clustering, we performed dimensionality reduction^37^ (Methods), which revealed cell clusters with broad distributions and some regions of overlap of neurons belonging to different e-types (Fig. 3d). A resampling analysis was performed to find an appropriate number of clusters and to establish robustness of the clustering (Extended Data Fig. 4). These analyses allowed to identify four distinct electrophysiological subtypes (e-types) of pyramidal neurons in L2–3 (Fig. 3c and Extended Data Fig. 4g). As a validation, we also used *k*-means clustering into *k* = 4 clusters with random seeds and found that it separated the pyramidal neurons into four very similar groups compared to hierarchical clustering (Extended Data Fig. 4h).

**Extended Data Fig. 5:**
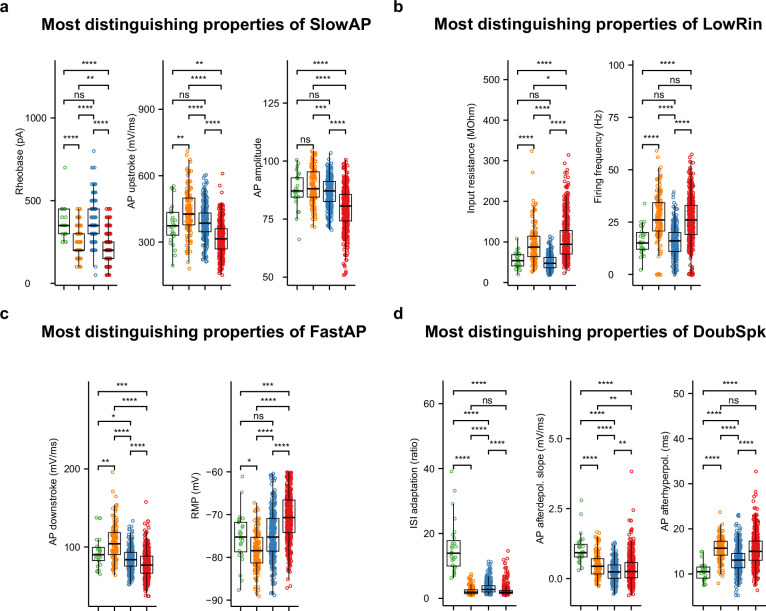
Pairwise comparisons between e-types for most distinguishing electrophysiological parameters. **a**–**d**, Box plots displaying, for SlowAP, LowRin, FastAP and DoubSpk e-types, respectively, their most distinguishing parameters in relation to all other e-types (color code, Fig. 3d; Methods). A plot of the parameter rheobase is omitted for LowRin neurons (**b**), and of AP upstroke for FastAP neurons (**d**), as these comparisons are already shown in preceding panels. Results of pairwise comparisons are depicted above the respective box plots (t-tests, Bonferroni-corrected, adjusted p-values are indicated by asterisks, * < 0.05, ** < 0.01, *** < 0.001, **** < 0.0001).

To better characterize the e-type clustering result in functional terms, we then identified the three most distinguishing features for each e-type according to their *F* values when comparing against all other neurons (Methods; Fig. 3e). More than one-third of neurons belonged to an e-type characterized by a low rheobase, comparatively slow AP upstroke and low AP amplitude that was termed ‘SlowAP’. In contrast, a ‘FastAP’ e-type exhibited faster AP upstroke and downstroke kinetics and more negative resting membrane potential. The ‘DoubSpk’ e-type represented a small group of neurons with initial doublet spiking, large interspike interval (ISI) adaptation, steep afterdepolarization slope and low AP afterhyperpolarization. Finally, the ‘LowRin’ e-type was overall less excitable and characterized by high rheobase, low input resistance and lower AP frequency. As expected, individual comparisons between e-types for the distinguishing variables were statistically significant in most cases (Extended Data Fig. 5).

**Extended Data Fig. 6:**
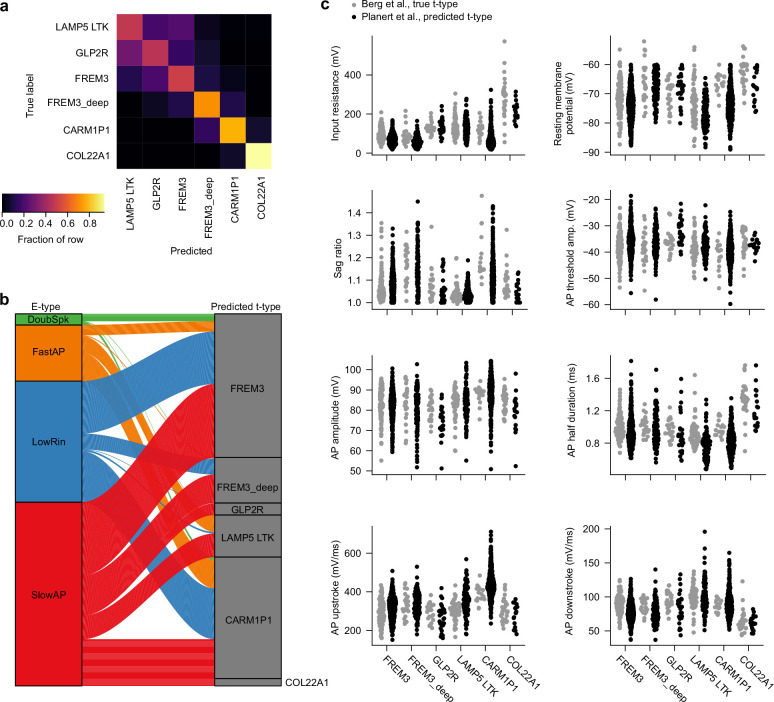
Correspondence analysis and assignment of e-types to pseudo-t-types. **a**, Class-balanced logistic regression classifier predicts t-types based on electrophysiological data^10^ using best-matching electrophysiological parameters between both studies (Methods). When tested on the original dataset^10^, the classifier had an accuracy of 64% for assigning the correct t-type label. **b**, Alluvial plot showing e-type to pseudo-t-type correspondence of n = 901 neurons of the core dataset of this study. **c**, Parameter distributions for the t-types in ref. ^10^ and pseudo-t-types assigned to the neurons in our study, demonstrating mostly similar distributions for each parameter and type.

A previous classification reported five transcriptomic subtypes (t-types) of pyramidal neurons in human L2–3 cortex^10^. To assess the correspondence to the e-types identified here, we built a logistic regression classifier based on eight matched electrophysiological parameters to predict their t-type labels (Methods). We first validated this classifier with the data of the original study and found the accuracy of t-type assignment to be 64% (Extended Data Fig. 6a). When applying the classifier to our electrophysiological dataset, neurons of all four e-types were assigned to all ‘pseudo-t-types’, except for the GLP2R and the COL22A1 groups. A total of 39% of all neurons were sorted into the superficial FREM t-type with large percentages from each e-type (67%, 18%, 43% and 40%, of ‘DoubSpk’, ‘FastAP’, ‘LowRin’ and ‘SlowAP’, respectively), suggesting that all e-types can resemble the electrophysiology of FREM neurons. The CARM1P1 pseudo-t-type group also consisted of neurons belonging to all four e-types. These results suggest no simple correspondence between t-types and e-types. Thus, our phenomenological classification represents an alternative and complementary approach toward subtype classification. Overall, our results support a functionally relevant subdivision of pyramidal neurons in upper L2–3 into four e-types, adding to the evidence that increased pyramidal neuron diversity is a general principle in human cortical L2–3.

### Functional subtypes exhibit distinct morphological features

**Fig. 4:**
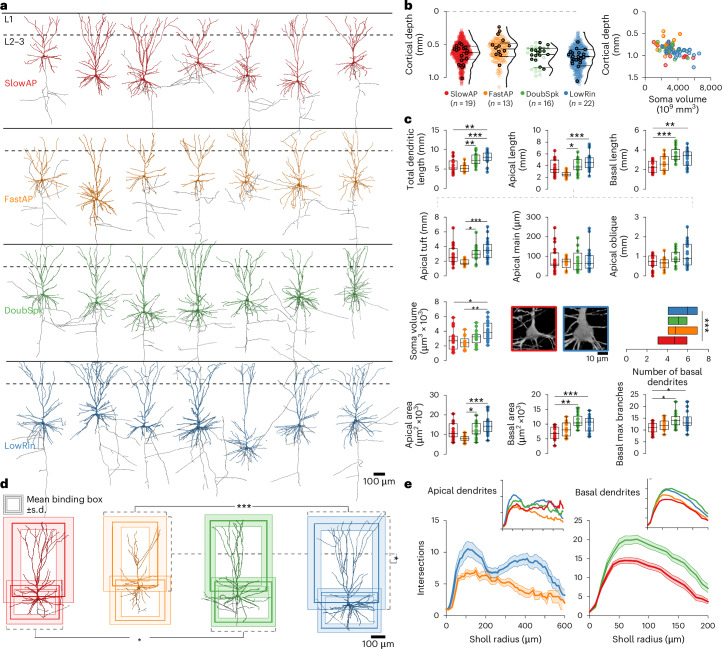
E-types are characterized by divergent morphometric features. **a**, Selection of reconstructed pyramidal neurons of the four e-types, aligned by cortical depth. Dendrites and somata are colored by e-type, axons shown in gray. **b**, Left: cortical depth distribution of all recorded (colored circles) and reconstructed neurons (circles with black outline) by e-types; horizontal ticks indicate median and IQRs. A total of 70 neurons were selected for reconstruction along the depth distribution of each e-type. Right: soma volume in relation to e-type (color coded) and cortical depth (correlation coefficient *R* = 0.49). **c**, Box plots of the total, apical and basal dendritic lengths, sample sizes indicated in **b** (Kruskal–Wallis test, post hoc Dunn’s multiple-comparison test, adjusted *P* values are indicated by asterisks, *P < 0.05, **P < 0.01, ***P < 0.001). A further subanalysis was performed for specific apical dendritic compartments, such as the apical tuft, apical main stem and length of oblique branches. E-types also exhibited distinct geometrical features as soma volume, surface area of basal and apical dendrites, and diverged in the number and maximum branch order of their basal dendrites. Means are depicted as centerlines, boxes indicate the IQR and whiskers extend to the smallest and largest values in the dataset. **d**, Binding boxes illustrate the vertical and horizontal spread (mean and s.d.) of the apical and basal dendrites of the four e-types (color coded; Kruskal–Wallis test, post hoc Dunn’s multiple-comparison test, adjusted *P* values are indicated by asterisks, *P < 0.05, ***P < 0.001). **e**, Sholl plots of the apical dendrites of LowRin (blue) versus FastAP cells (orange, left) and of the basal dendrites of DoubSpk (green) versus SlowAP cells (red, right). Shaded areas depict the s.e.m.; insets illustrate average Sholl profiles of all e-types.

Morphological diversity has been described for human L2–3 pyramidal neurons^10,14,17,24,38,39^. We, therefore, asked how our e-type classification is paralleled by differences in neuroanatomical characteristics. To address this question, we created full three-dimensional reconstructions from confocal image stacks of a subset of the recorded and visualized pyramidal neurons (70 neurons; Fig. 4a) and analyzed their dendritic and axonal morphologies. While peaks of the depth distributions showed some divergence between the e-types, individual neurons were scattered widely over most of the depth of L2–3, with large overlap between e-types and a moderate increase of soma volume with depth across e-types (Fig. 4b). All reconstructed neurons were identified as pyramidal neurons based on their dendritic morphology (Methods). In primates, the axon primarily emerges from the soma^40^. In our study, we observed the emergence of the axon from the base of the soma in all cases examined (*n* = 70/70). Axons extended vertically toward the white matter, giving rise to sparsely distributed collaterals with horizontal orientation. The neurons showed differences in their dendritic arborization patterns and size increasing with cortical depth, in line with the previous studies^10,14,41^. However, dendritic length showed a moderate correlation with their distance from the pia (*R* = 0.42), suggesting that additional factors contribute to their heterogeneity.

**Extended Data Fig. 7:**
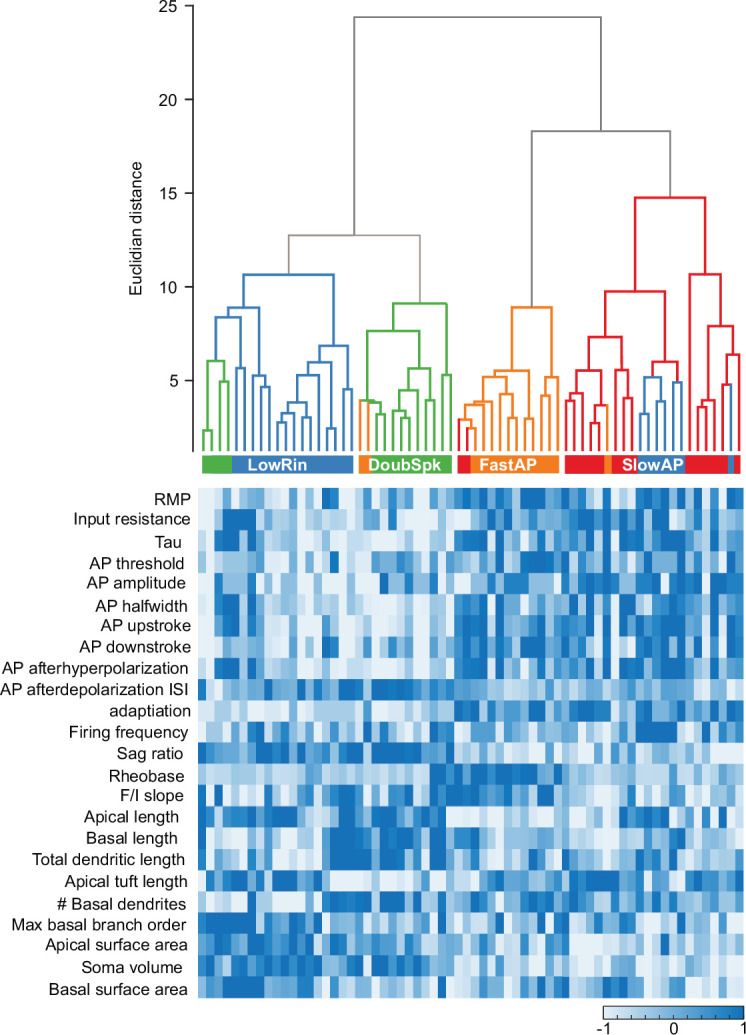
Cluster analysis including both anatomical and electrophysiological parameters. Dendrogram showing the result of hierarchical clustering of the n = 66 pyramidal neurons that were both included in the core dataset for cellular analysis (Extended Data Fig. 1a) and had extended anatomical information. The cluster analysis included 9 morphological and 15 electrophysiological parameters (lower panel, each row represents one parameter and each column represents one neuron). The results and association with e-types are shown in the top panel. Due to relatively low n no statistical tests were performed for cluster separation, but according to the results, either three or four me-clusters or -types can be hypothesized. Generally, most of the neurons belonging to a specific e-type were sorted in the same me-cluster (see results text for more details).

To assess whether clustering based on electrophysiological features also reflects differences among morphoelectric subtypes (me-types), we repeated the cluster analysis, including both morphological and electrophysiological parameters. We included all high-resolution reconstructions of pyramidal neurons that were part of the core dataset of cellular property analysis (*n* = 66), and we quantified nine commonly assessed morphological parameters^10^ (Methods). Generally, most of the neurons belonging to a specific e-type were sorted to the same cluster or me-type (Extended Data Fig. 7). When assuming four me-types, only 19 of 66 neurons were sorted to a different cluster than most of their e-type, and thus 71.2% of cells had a matching assigned e-type. When assuming three me-types (with the uncommon DoubSpk e-type being sorted together with LowRin), only 12 of 66 neurons were sorted outside of their main cluster (81.8% within the reference group). These results demonstrate considerable consistency between the morphoelectric signature and the e-type clustering.

To confirm that e-types exhibit corresponding differences in their morphological parameters, we separated and compared their morphology (Fig. 4c–e). The LowRin type had the longest total dendritic length and the FastAP type had the shortest total dendritic length (Fig. 4c). This difference was also evident in the length of the apical dendrites and could be attributed to a difference in the apical tuft size that was further supported by differences in horizontal spread of dendrites (Fig. 4c,d). Consistent with their depth distribution and the low input resistance of LowRin neurons, the difference in apical tuft size across e-types was accompanied by similar differences in soma volume (Fig. 4c) and in the vertical spread of dendrites (Fig. 4d). Sholl analysis of dendrites showed the highest degree of branching complexity of the apical dendrites in LowRin with two peaks corresponding to the domain of oblique side branches and to the tuft (Fig. 4e). In contrast, FastAP neurons had the lowest level of branching over the full extent of their apical dendrites (Fig. 4e). DoubSpk and SlowAP cells displayed marked differences in their basal dendrite morphology, with DoubSpk having the longest basal dendrites when compared to SlowAP cells (Fig. 4c). The DoubSpk type also displayed the highest and SlowAP type the lowest branching of the basal dendrites in Sholl analysis (Fig. 4e). In summary, we found substantial quantitative differences in the somatic and dendritic structure of e-types. The results corroborate our e-type classification and suggest that it represents distinct morphoelectric subtypes of L2–3 pyramidal neurons in the human temporal cortex.

### Connectivity and synaptic properties are subtype-specific with LowRin receiving frequent inputs, while SlowAP forms sparse, strong and depressing synapses

**Fig. 5:**
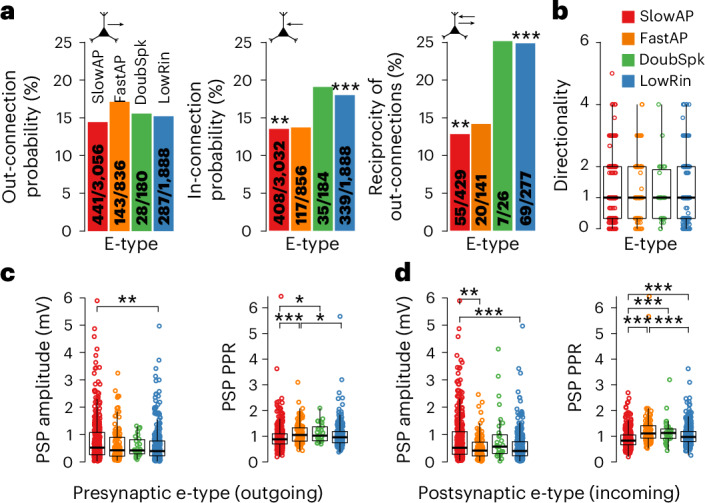
Incoming and outgoing connectivity and synaptic function according to e-type. **a**, Bar graphs depicting connection probability according to presynaptic (left) or postsynaptic e-type (middle), and reciprocity of positive outgoing connections tested in both directions (right). Numbers represent found and tested connections and numbers of reciprocal connections for bidirectionally tested pairs. Middle and right: chi-squared tests for effect of e-type were statistically significant (*X*^2^ = 21.991, df = 3, *P* < 0.001; *X*^2^ = 19.871, df = 3, *P* < 0.001, respectively). Bonferroni-corrected *P* values of difference from expected probability in post hoc tests are shown by asterisks, **P < 0.01, ***P< 0.001. **b**, Directionality, that is, the ‘feed-forwardness’ of connectivity, is shown for each e-type at the neuron level (SlowAP, *n* = 421; FastAP, *n* = 121; DoubSpk, *n* = 27; LowRin, *n* = 254), calculated by the squared difference between in-connections and out-connections normalized by the number of total connections of each neuron^31,64^ (*P* = 0.24 in Kruskal–Wallis rank-sum test). **c**,**d**, Scatterplots and box plots show EPSP amplitude and PPR depending on presynaptic or postsynaptic e-type. Number of out-connections/in-connections of presynaptic/postsynaptic neuron, respectively—SlowAP, *n* = 422 and 399; FastAP, *n* = 133 and 110; DoubSpk, *n* = 26 and 32; LowRin, *n* = 285 and 325. All *P* values in Kruskal–Wallis rank-sum tests for data depicted in each panel were *P* < 0.01. Asterisks indicate, for each panel, adjusted *P* values of post hoc Dunn’s tests. All *P* values are indicated by asterisks, *P < 0.05, **P < 0.01, ***P < 0.001. Medians are shown as centerlines, boxes indicate the IQR, whiskers extend to values within 1.5 × IQR and outliers are plotted individually.

Having established an electromorphological classification of pyramidal neurons in L2–3, we used it as a tool to resolve whether e-types have distinct connectivity and synaptic properties. We first investigated whether synapses differed depending on e-type of the presynaptic or postsynaptic neuron (Fig. 5). Generally, out-connectivity did not, but in-connectivity differed according to e-type (Fig. 5a). LowRin neurons received more, while SlowAP neurons received less synaptic inputs than expected when assuming uniform distribution across subtypes. In a separate study on this dataset, we showed that increased directionality and random reciprocity are distinct features of human L2–3 pyramidal neurons^31^. While reciprocity was higher in LowRin and lower in SlowAP when considered as presynaptic neurons (Fig. 5a), we found no significant difference in directionality between e-types on the individual neuron level (Fig. 5b). We then investigated synaptic properties and found that outgoing and incoming EPSPs of SlowAP neurons were larger in amplitude and had more depressing short-term plasticity compared to other subtypes (Fig. 5c,d). LowRin neurons showed high in-connectivity with synapses of smaller initial amplitude and stronger facilitation compared to SlowAP, whereas SlowAP neurons received fewer, but stronger incoming synapses. In summary, both connectivity and EPSP properties varied by e-type.

**Fig. 6:**
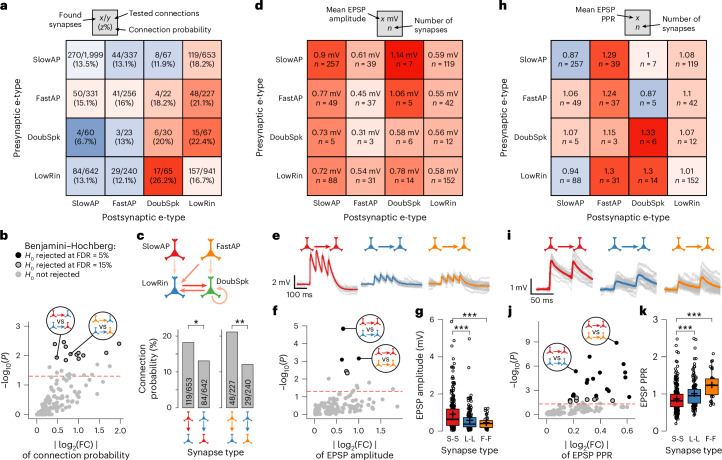
Specific connectivity and synaptic function according to synapse type. **a**, Matrix color coded by connection probabilities for all 16 synapse types (combination of presynaptic and postsynaptic e-types). **b**, Volcano plot of relative connectivity (FC, *x* axis) for 120 comparisons between the 16 synapse types versus statistical significance (*y* axis). Comparisons above the red dashed line are significant at *P* < 0.05. Gray dots with black circles indicate significant comparisons after multiple-comparison correction at FDR of 15% (Benjamini–Hochberg). Dots with circled insets indicate comparisons exemplified in the bar graph of **c**. **c**, Top: schematic of wiring principle between e-types. Arrow colors represent connection probability, as shown in **a**, connections of <18% are not shown. Bottom: bar graphs comparing connection probabilities between SlowAP (red) and LowRin (blue), and between FastAP (orange) and LowRin (blue), with the opposite direction, respectively (Fisher’s exact test, *P* values are indicated by asterisks, *P < 0.05, **P < 0.01). **d**, Matrix color coded by the mean EPSP amplitude for all 16 synapse types. **e**, EPSP traces from representative homotypic synapse types. Gray traces are single sweeps; thick colored line shows the average across sweeps. **f**, Volcano plot as in **b** for EPSP amplitude. Black dots indicate comparisons that remain significant after correction with an FDR of 5%. **g**, Jitter and box plots show the EPSP amplitudes of homotypic synapse types of SlowAP (red), LowRin (blue) and FastAP (orange) neurons (Mann–Whitney *U* test, two-tailed; sample sizes shown in **d**; cross-symbols indicate mean). **h**–**k**, Same as in **d**–**g** but for EPSP PPR. *P* values below 0.05 are indicated by asterisks, ***P < 0.001. Medians are shown as centerlines, boxes indicate the IQR, whiskers extend to values within 1.5 × IQR and outliers are plotted individually. FC, fold change.

**Extended Data Fig. 8:**
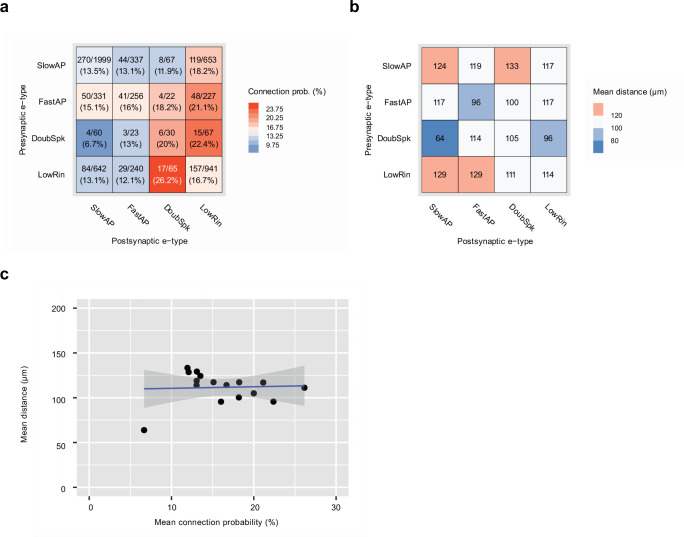
Relationship between distance and connection probability of different synapse types. **a**, Connectivity for each synapse type (as in Fig. 6a, including number of positive/number of tested connections and connection probability in brackets). **b**, Mean intersomatic distance for each synapse type. **c**, Connectivity of each synapse type plotted against their mean distance, including regression line (and 95% confidence interval, CI). The correlation revealed no strong relationship between the two measures (Pearson’s r = 0.0486). Fitting a regression model to the data resulted in a multiple R^2^ of 0.002 and p = 0.85, thus modeling average connectivity by average distance provided no better fit than a model without any predictor. Mean connectivity between DoubSpk and SlowAP neurons was very low in our sample (6.7%), as was mean distance (64 µM). This stands in contrast to the general finding of no relationship between distance and connectivity; however, the sample is comparatively small for this combination (4 positive out of 60 tested connections).

**Extended Data Fig. 9:**
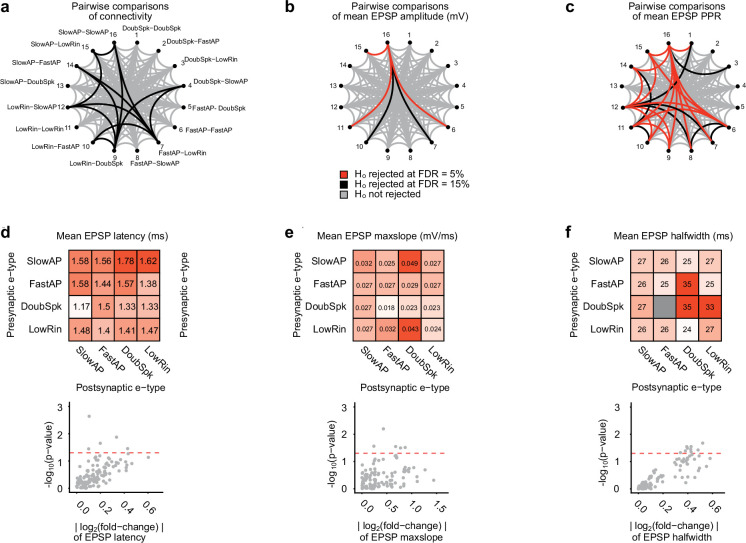
E-type specific synaptic function. **a**–**c**, All possible comparisons of e-type pairs for connectivity, mean EPSP amplitude and mean EPSP paired pulse ratio corresponding to the respective volcano plots shown in Fig. 6. Connection lines for comparisons for which null hypothesis was rejected, after correcting for multiple comparisons using B&H method with false discovery rate (FDR) of 15% and 5%, are depicted in black and red, respectively. **d**–**f**, Top row, matrix color coded by mean EPSP latency, maximum slope and halfwidth, respectively, for all 16 synapse types (total n of each parameter: see Extended Data Fig. 1a). Bottom row, volcano plots for 120 pairwise comparisons of synapse types for the respective synaptic parameter. Relative difference in mean (fold-change) for 120 pairwise comparisons of synapse types plotted against their level of statistical significance (Mann-Whitney *U* test). Some comparisons resulted in a p-value < 0.05 (dots above red dashed line), but no comparison was significant at FDR of 15%.

To examine microcircuits at the synapse level in relation to e-type, we classified all connected and probed neuron pairs into 16 synapse types, defined by the combination of presynaptic and postsynaptic e-type. Connection probability across synapse types varied from 7% to 26% (Fig. 6a). To control for differences in average soma distance underlying differential connectivity, we used a regression model and found only weak correlation (Pearson’s *r* = 0.05), and no significant contribution of somatic distance to mean connectivity of synapse types (multiple *R*^2^ = 0.002, *P* = 0.85; Extended Data Fig. 8). Connections between DoubSpk and LowRin had the highest connection probabilities of all synapse types (22.4% and 26.2%; Fig. 6a). Consistent with the neuron-level connectivity analysis (Fig. 5a), LowRin neurons also received many connections from SlowAP (18.2%, 119 of 653) and FastAP neurons (21.1%, 48 of 227), whereas connections from LowRin back onto SlowAP (13.1%, 84 of 642) and FastAP neurons (12.1%, 29 of 240) were significantly less frequent (Fig. 6a,c). Systematic comparison of all 120 possible combinations of the 16 synapse types revealed 12 statistically significant differences at a false discovery rate (FDR) of 15% (Benjamini–Hochberg method^42^; Fig. 6b). While approximately two of these differences are expected to be false positives, in 7 of the 12 statistically significant comparisons, connectivity was higher when the postsynaptic neuron was LowRin (Extended Data Fig. 9a). In contrast, in 8 of the 12 significant comparisons, connectivity was lower when the postsynaptic neuron was SlowAP (Extended Data Fig. 9a). These examples of e-type-specific connectivity indicate that LowRin neurons preferentially receive more inputs from other e-types rather than relaying information to them.

We then compared EPSPs across all synapse types. These EPSPs were measured at the soma and represented summarized responses of potentially multiple anatomical synaptic contact sites^43^. Mean EPSP amplitudes varied between 0.31 and 1.14 mV (Fig. 6d). Systematic comparison of all 120 possible combinations of the 16 synapse types revealed three statistically significant differences of mean EPSP amplitude at an FDR of 5% (Fig. 6f and Extended Data Fig. 9b). The largest EPSP amplitudes included homotypic connections, that is, connections between neurons of the same e-type (Fig. 6d), specifically between SlowAP. These were significantly stronger than homotypic connections of LowRin neurons (0.9 mV versus 0.58 mV; Mann–Whitney *U* test, *P* < 0.001) and FastAP neurons (0.45 mV; *P* < 0.001; Fig. 6e–g). EPSP PPR differed widely across synapse types, ranging from 0.87 to 1.33 (Fig. 6h), and we identified 17 significant comparisons at an FDR of 5% (Fig. 6j). In accordance with the amplitude results, homotypic connections between SlowAP neurons exhibited stronger paired pulse depression (0.87) compared to connections between LowRin (1.01; *P* < 0.001), and to mainly facilitating connections between FastAP neurons (1.24; *P* < 0.001; Fig. 6i–k). We performed analogous analyses for the remaining synaptic parameters (EPSP latency, maximum slope and halfwidth; Extended Data Fig. 9) but did not identify significant differences. Taken together, we found e-type-specific differences in a subset of synaptic properties, including strong homotypic connectivity of SlowAP, and a high degree of specification in short-term plasticity.

Overall, our detailed analysis at the synapse level uncovered substantial heterogeneity in connectivity and synaptic function across e-types (Figs. 5 and 6), revealing differential functional embedding of L2–3 pyramidal neuron subtypes in the excitatory microcircuit. However, the observed variability in synaptic properties (Fig. 2f,g) exceeded the mean difference between functionally defined pyramidal neuron subtypes (Figs. 5 and 6), indicating that functional classification alone cannot account for the full diversity. This extensive synaptic diversity suggests the existence of additional mechanisms that drive synaptic specialization.

### Principles of L2–3 microcircuit organization are conserved across individual participants

**Fig. 7:**
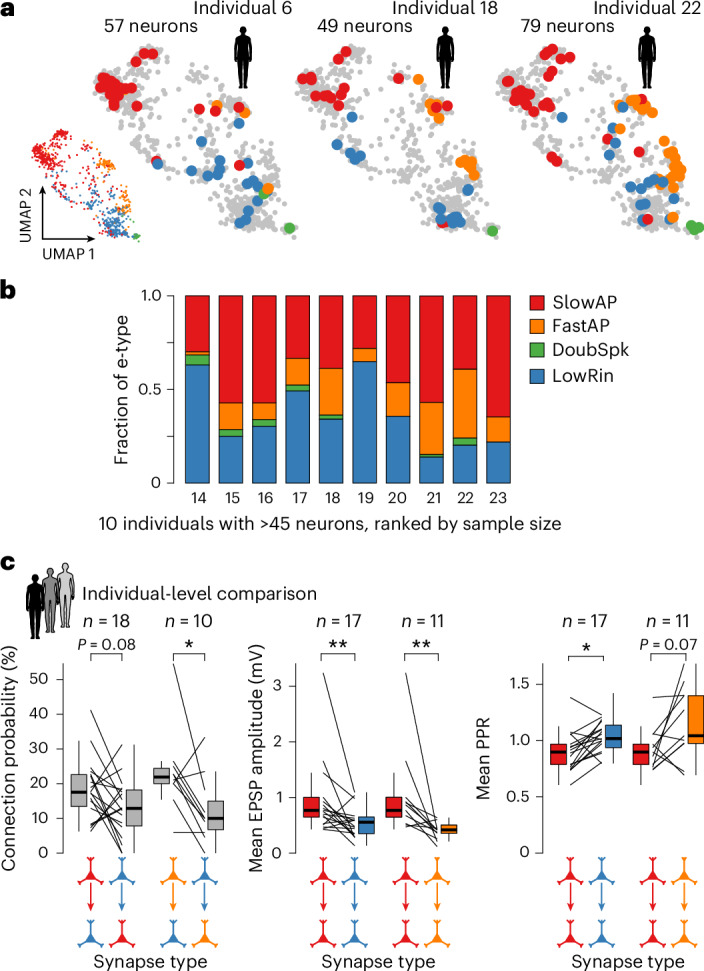
Conserved principles of microcircuit organization of L2–3 across human individuals. **a**, UMAP visualization of the high-dimensional parameter space as in Fig. 3d, with each dot representing one neuron color coded by assigned e-type (bottom left), and three separate UMAPs showing in color the neurons recorded from selected individuals with large sample sizes. The numbers assigned to the example individuals (Extended Data Fig. 1b) are shown above the schematic human body images. Note that all four e-types are present in each of the example individuals. **b**, Stacked bar plot shows relative fraction of e-types for ten patients, for which more than 45 pyramidal neurons were recorded. Numbers assigned to each individual are shown below the stacked bar. **c**, Bar graphs compare connection probabilities, EPSP amplitudes and PPR between synapse types that were statistically significant at the neuron/synapse level (Fig. 6c), now plotted and tested at the individual level. Each line represents one human participant; participants with less than ten tested connections for one of the synapse types were excluded (Wilcoxon signed rank-sum test. *P* values of <0.05 are indicated by asterisks, *P < 0.05, **P < 0.01). Medians are shown as centerlines, boxes indicate the IQR and whiskers extend to values within 1.5 × IQR.

Differential microcircuit embedding of pyramidal neuron subtypes suggests a general principle in which e-types serve as functional determinants of cortical computation. We thus hypothesized that e-types and their specific connectivity patterns would be conserved within and across individuals. UMAPs of individuals with high numbers of recorded neurons showed similar patterns of distribution of e-types within the parameter space (Fig. 7a). While individuals with more than 45 neurons recorded contain the three most common e-types, only the rare DoubSpk e-type was sometimes not sampled (3/10; Fig. 7b). When comparing patient averages, connectivity preferences between FastAP and LowRin, as well as differential synaptic properties of homotypic synapses (Fig. 6c), were preserved: while connection probability from SlowAP to LowRin neurons failed to reach significance in comparison to the other direction (*P* = 0.07), the higher connectivity from FastAP onto LowRin compared to the reverse direction was statistically significant (Fig. 7c). Also, most differences between strong and depressing synapses of homotypic SlowAP versus weaker and more facilitating synapses for homotypic LowRin synapses remained significant at the individual patient level and support our hypothesis that e-type-specific connectivity principles are generalizable across individuals.

## Discussion

Here we identify principles of subtype-specific synaptic properties and local network organization of human temporal cortex that are preserved across individuals. By recording over 1,400 pyramidal neurons in L2–3, we were able to characterize four electrophysiological subtypes. Previous studies suggested five to ten molecularly identified subtypes in human L2–3 (refs. ^10,11,15^). While in L5 the main t-type and e-type overlap^36^, the predictability of L2–3 t-types based on electrophysiological properties is limited^10^. In line with this, our correspondence analysis (Extended Data Fig. 6) revealed a low specificity of e-type to pseudo-t-type mapping. This could arise from the fact that protein enrichment in the brain can be discordant with gene expression^44^. Given that the physiology of a neuron is substantially determined by post-translational regulations, a phenotypic subclassification provides a more direct understanding of microcircuit computation. E-types may represent functional states or gradients across t-types in L2–3 of human temporal cortex^35^. Future studies that combine molecular analysis with synaptic physiology are needed to resolve the role of transcriptomic subtypes in local connectivity.

Our data capture the skewed distribution of synaptic amplitudes and PPR within individual human participants (Fig. 2 and Extended Data Fig. 3). log normally distributed amplitudes with many weak and few strong synapses have been reported in excitatory and inhibitory synapses across brain regions and species, and are likely important for network computations and plasticity^25,26,45–47^. While previous studies reported paired pulse depression at higher stimulation frequencies^25,32^, intermediate stimulation frequency allowed to establish a log–log relationship of amplitude and PPR, unmasking short-term facilitation of weak synapses. This power–law relationship provides a parameter range spanning up to two orders of magnitude (for EPSP amplitude) supporting enriched computations.

We observed morphological differences in the somatodendritic domain of e-types (Fig. 4). Previous studies have shown that human L2–3 pyramidal neurons have increased dendritic length and complexity compared to rodents^41^, and that these features are strongly expressed in the human temporal cortex^38^. A classification into slim-tufted and profuse-tufted pyramidal cells has also been proposed^14^. This study was based on the apical branching density and reported slim-tufted neurons at greater cortical depths, while our analysis focused on dendritic length in upper L2–3, making a direct comparison difficult. Our findings extend prior anatomical work by linking morphology to synaptic physiology. Specifically, we show that LowRin neurons, with their longer basal dendrites, exhibit higher in-connectivity than SlowAP neurons, while their larger soma size is paralleled by lower input resistance and smaller EPSP amplitudes. These results suggest that morphology can account for some differences in synaptic function between e-types, but additional mechanisms likely underlie the full diversity of synaptic function.

The differences across e-types in dendritic morphology, connectivity and synaptic function, together with their cellular physiologies, suggest distinct roles of pyramidal neuron subtypes in microcircuit computation. This principle of subtype-specific computation is paralleled by the functional heterogeneity of projection neurons observed in the rodent hippocampus^48,49^. For example, the LowRin subtype has high input connectivity and complex apical dendrites, a characteristic feature of human association cortices linked to higher cognitive function^50–52^. Together with its lower excitability, it appears especially suited to integrate information locally. We further identified DoubSpk neurons with complex basal dendrites that are frequently connected to LowRin. Physiologically, they resemble burst firing subtypes in human deep L3 and L5 (refs. ^10,17,53^) and may serve facilitated information transfer^54,55^. At the synaptic level, we observed a large dynamic range of synaptic amplitudes and short-term plasticity, which, according to network models, can enhance computation^56^. Specifically, PPR differed widely depending on presynaptic and postsynaptic e-type combination. While connections between SlowAP exhibited predominantly paired pulse depression, connections between FastAP were strongly facilitating. As synaptic dynamics implement important filter functions for incoming connections^57^, our findings suggest subtype-specific information processing. Taken together, our study demonstrates that increased neuronal diversity is accompanied by synaptic specialization. Together with other principles of human microcircuit organization^31,58^, the subnetwork complexity found here likely supports advanced computations in human cortical pyramidal neuron networks.

## Limitations

**Extended Data Fig. 10:**
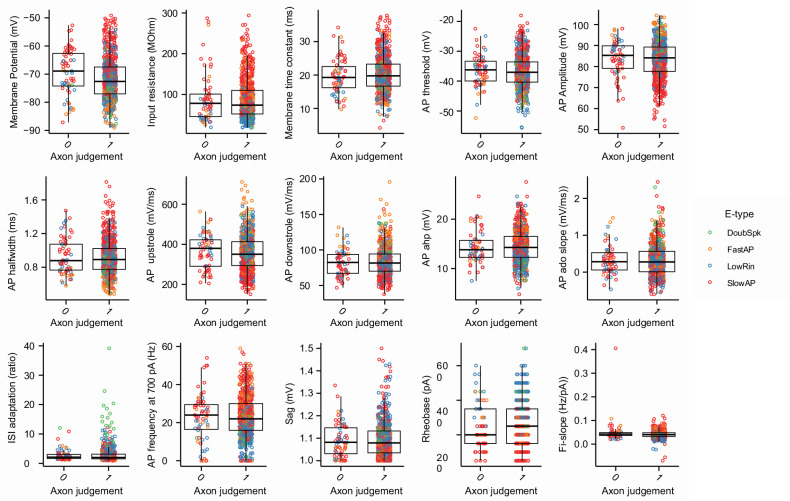
Early axon truncation effects on electrophysiological properties. For pyramidal neurons from the dataset for which detailed axon judgment information was available and that had an associated e-type (color code, total n = 722), box plots depict cellular properties of the neurons with early truncated axons (and no previous collateral, n = 63) on the left, and those that had a positive axon judgment for connectivity analysis (n = 659, see Methods) on the right. Intrinsic properties did not differ significantly between these groups (unadjusted p-values all > 0.05, t-tests). E-types were generally found across groups.

Studying functional heterogeneity in acute slices comes with the limitation of possible effects of truncation of axons and dendrites. We implemented several steps of data curation and quality control to minimize underestimation of connectivity and changes in intrinsic properties (Methods; Extended Data Fig. 10). The validity of this approach is indicated by a slightly higher local connection probability compared to previous human multipatch studies (16% versus ~13%; Fig. 2e (refs. ^25–27,29,30^)). Furthermore, a cluster- and neuron-level analysis of truncation effects on connectivity within a separate study on this dataset showed that heterogeneity of connectivity is intrinsic to the microcircuit and not due to slice cutting artifacts^31^.

Like all neurophysiological studies on acute human cortical slices^10,17,41,53^, we used resected tissue from neurosurgical patients. To reduce variability due to subregion specificity and disease entity^15,23,39,59^, we confined our study to patients with pharmaco-resistant epilepsy that underwent anterior temporal lobe resection surgery, and to upper portions of L2–3 in one defined area. We excluded structural abnormalities through neuropathological assessment. While cellular and synaptic variability may still be associated with epilepsy or its underlying etiology^60–62^, associations of epilepsy-related disease parameters with patient averages of cellular properties were not significant after correcting for multiple comparisons (Extended Data Fig. 2). Despite substantial differences with regard to sex, age and patient phenotype, we found conserved principles of cellular diversity and synaptic connectivity across individuals.

## Methods

### Experimental procedures

The study procedures adhered to all relevant ethical regulations and were approved by the local ethical committee (Ethikkommission der Charité—Universitätsmedizin Berlin) with approval EA2/111/14. Data collection and analysis were not performed blindly to the experimental conditions.

#### Human participants

Prior written informed consent for the scientific use of resected tissue was given by the patients. The data obtained from this study were measured in acute brain slices obtained from temporal lobe resections in 23 patients with drug-resistant epilepsy (12 male, 11 female; age range = 21–55 years, median age = 34 years).

#### Human brain slice preparation

Temporal lobe pole tissue resected from the patients was transferred from the operating theater to the laboratory in sterile and cooled sucrose-based artificial cerebrospinal fluid (aCSF; 87 mM NaCl, 2.5 mM KCl, 3 mM MgCl_2_, 0.5 mM CaCl_2_, 10 mM glucose, 75 mM sucrose, 1.25 mM NaH_2_PO_4_ and 25 mM NaHCO_3_, 310 mOsm) enriched with carbogen (95% O_2_, 5% CO_2_) within 15–40 min after resection. The arachnoid tissue was removed from the surface and the tissue was cut under sterile conditions in ice-cold sucrose-based aCSF to 300- or 400-µm-thick slices. After a 30-min recovery period at 34 °C, slices were held submerged at room temperature in sucrose-based aCSF. In a subset of experiments, an antibiotic (Minocycline, 2 µM) was added to the incubation solution. Recordings were made up to 52 h after slicing (interquartile range = 7.5–26 h).

#### Multineuron patch-clamp recordings

Slices were transferred to the recording chambers of two patch-clamp setups optimized for high-throughput microcircuit assessment of rare tissue samples, including automated pipette pressure and pipette cleaning systems^30^. The eight-manipulator setup included the following equipment: eight PatchStar manipulators (Scientifica), BX-51 WI microscope (Olympus), Orca-Flash 4.0 LT camera (Hamamatsu Photonics), MultiClamp 700B amplifiers (Molecular Devices) and Digidata 1550 digitizer (Molecular Devices). The ten-manipulator setup included ten u-Mp micromanipulators (Sensapex), Eclipse FN1 microscope (Nikon), Orca-Flash 4.0 LT camera (Hamamatsu Photonics), MultiClamp 700B amplifier (Molecular Devices) and Power1401-3A digitizer (Cambridge Electronic Design). Data were low-pass filtered with Bessel filter at 6 kHz and digitized at a sampling rate of 20 kHz (with a small subset of data sampled at 10 kHz). Data acquisition was performed using pClamp 10 (Molecular Devices) or Signal 6 acquisition software (Cambridge Electronic Design).

Patch pipettes were pulled from borosilicate glass capillaries (2 mm outer/1 mm inner diameter; Hilgenberg) on a horizontal puller (P-97, Sutter Instrument). They were filled with K-gluconate-based intracellular solution containing 130 mM K-gluconate, 2 mM MgCl_2_, 0.2 mM EGTA, 10 mM Na_2_-phosphocreatine, 2 mM Na_2_ATP, 0.5 mM Na_2_GTP, 10 mM HEPES buffer and 0.1% (w/v) biocytin (290–295 mOsm, pH adjusted to 7.2 with KOH) and had 5–8 MΩ resistance. The liquid junction potential was calculated to be 16 mV and was not corrected for. Neurons were visualized using the differential interference contrast infrared microscope and pyramidal neuron-like soma was selected. To target L2–3, we only included neurons that were recorded at a cortical depth up to 1,200 µm (distance from pia). To allow for within-individual and between-individual analyses, we only included data from individuals in which we recorded more than 25 neurons. Whole-cell patch-clamp recordings of these L2–3 neurons were performed at 34 °C under submerged conditions, with continuous perfusion of aCSF containing 125 mM NaCl, 2.5 mM KCl, 1 mM MgCl_2_, 2 mM CaCl_2_, 10 mM glucose, 1.25 mM NaH_2_PO_4_ and 25 mM NaHCO_3_ (300 mOsm). Pipette access resistance and capacitance were compensated throughout the experiment, and the remaining effects of these were addressed offline using reverse resistor–capacitor filtering (‘Quality control’ and ‘Defiltering of AP traces’). In some cases of recording failure, we repatched neurons with a cleaned pipette using our automated ‘clean2complete’ approach^30^. For the assessment of cellular and synaptic electrophysiological properties, we applied various stimulation protocols in voltage and current clamp (see below).

#### Morphological reconstruction

After recording, slices were fixed overnight in a fixative solution containing 4% paraformaldehyde with 0.1 M PBS (pH 7.4) at 4 °C for 24–48 h. The biocytin-filled neurons were subsequently visualized using avidin conjugated fluorochrome (Alexa Fluor-647, Invitrogen; 1:1,000) in a PBS (0.9% NaCl) solution containing 3% NGS, 0.1% Triton X-100 and 0.05% NaN_3_ for 24 h at 4 °C. Slices were rinsed in PBS before being mounted in aqueous medium (Fluoromount-G, Southern Biotech) between cover slips with a 300-μm-thick metal spacer to prevent shrinkage^65^. Visualized neurons were imaged on a laser scanning confocal microscope (FV1000, Olympus) for morphological identification. Selected neurons were reconstructed using the Neutube software package^66^ from composite image stacks obtained with a 30× silicone immersion objective (N.A. 1.05, UPlanSApo, Olympus) over the full extent of the cells.

#### Neuropathological assessment

To confirm cortical tissue quality, parts of the temporal lobe pole tissue, including slices from brain slice preparation, were selected for histopathological evaluation by a board-certified neuropathologist. The analyzed slice samples revealed no structural changes. A subset of tissue samples of two patients exhibited epilepsy-associated pathology (low-grade glioneuronal tumor and focal cortical dysplasia) or micro-infarcts. All these pathologies were located in the temporal lobe, but not in the tissue used for the slices examined. Patch-clamp recordings of these three patients also displayed no obvious differences in cellular electrophysiology. This, together with the observation that ~75% of the anatomically analyzed neurons in the dataset exhibited typical pyramidal neuron morphology (see below), suggests that the recorded pyramidal neuron properties were not affected by pathology.

### Cellular physiology analysis

We developed custom MATLAB code to perform automated analysis of recorded raw traces, comparable to previous studies^26,67^, as outlined below.

#### Resting membrane potential, input resistance, membrane time constant and sag ratio

To estimate the ‘resting membrane potential’, we took average amplitudes of four 150-ms trace segments, located before the –50, 50, 100 and 150-pA step current injections. The mean of the four values was used as the final ‘resting membrane potential’. The ‘input resistance’ was calculated as the difference between the steady-state voltage and the ‘resting membrane potential’, divided by the injected current, using –100, –50, 50, 100-pA current steps. The average voltage of the last 100 ms before the end of the step current stimulus was defined as the steady state^67^. The mean of the ‘input resistance’ values at the different step current injections was used as the final ‘input resistance’. To estimate the ‘membrane time constant’, the *τ* values of mono-exponential functions (equation (1)), which were fit to the initial segments of the voltage responses to the −50 and −100-pA step currents, were used. We used the mean of the two *τ* values as the ‘membrane time constant’.

1 $${{ae}}^{\frac{-x}{\tau }}+b$$

To approximate the sag, we fit the voltage response to a –100-pA step current with a function of the form (equation (2)). The sag ratio was calculated from the model fit as the ratio of the negative peak voltage amplitude measured from the resting membrane potential to the respective amplitude of the steady-state voltage during current injection.

2 $${e}^{\frac{-\left(x-c\right)}{{\tau }^{1}}}-\left({{ae}}^{\frac{-\left(x-c\right)}{{\tau }^{2}}}+{{be}}^{\frac{-\left(x-c\right)}{{\tau }^{3}}}\right)-d$$

#### Defiltering of AP traces

APs are fast signals and thus prone to filtering artifacts. Because it is not possible to compensate the entire capacitance of the patch-clamp pipette in current clamp mode, its remainder (referred to as parasitic capacitance^68^), combined with the access resistance, forms a filter. Some AP parameters extracted from the original traces show a correlation with the access resistance due to an increasing effect of this filter with larger access resistance (Extended Data Fig. 1). The AP upstroke and the AP amplitude are mostly affected (Pearson correlation coefficient is −0.6 for both correlations). Even if one sets strict limits for the access resistance of included recordings, this filter artifact will still affect AP-shape parameters recorded with low access resistance. Because we measured the access resistance and can estimate the parasitic capacitance, we can hence approximate the effect of the filtering. In an approach similar to the one mentioned in ref. ^69^, we used an inverse digital resistor–capacitor-filter algorithm to produce a defiltered AP signal for each neuron (Extended Data Fig. 1c). The parameters extracted from the defiltered signals show a much weaker correlation with the access resistance. Correcting for the dependence of parameters on the access resistance allowed further analysis with minimized technical bias.

#### AP parameters

AP parameters were extracted from the first AP elicited in response to a series of increasing step current injections (increments of 50 pA). All measurements were taken from the defiltered trace. The ‘AP threshold amplitude’ was measured on the ascending trace where the slope exceeded 20 mV ms^−1^. The maximum and the minimum of the AP slope were defined as the ‘AP upstroke’ and ‘downstroke’, respectively. The ‘AP amplitude’ was measured from the ‘AP threshold’ to the maximum peak. The ‘AP halfwidth’ is the width of the AP (in milliseconds) at half AP ‘amplitude’. The ‘AP afterhyperpolarization’ was measured as the difference between the ‘AP threshold’ and the minimum after the AP. If no minimum was reached in a 10.0-ms time window, the amplitude at the point after the AP, where the slope exceeded −0.2 mV ms^−1^, was used instead. The ‘AP afterdepolarization slope’ was defined as the mean slope of the voltage trace in a 1.0-ms time window after the ‘AP afterhyperpolarization’.

#### Firing properties

To characterize the firing properties of a neuron, the ‘rheobase, firing frequency at 700 pA, frequency/current slope (*F*/*I* slope)’ and the ‘interspike interval adaptation index (ISI adaptation)’ were extracted. The first step, the current stimulus that elicited at least one AP, is referred to as the ‘rheobase’. The ‘firing frequency at 700 pA’ is the frequency of APs elicited in response to a 700-pA step. The ‘*F*/*I* slope’ is the slope of a linear fit through the firing frequencies at increasing step current amplitudes (50–100-pA increments). To calculate the ‘ISI adaptation’, the first five step currents that elicited at least three APs were used^67^. The ‘ISI adaptation’ was calculated separately for each current injection by dividing the second ISI (interval between second and third AP) by the first (interval between the first and second AP). The mean value of those five ratios was taken as the final ‘ISI adaptation’ (if less than five values were available, the mean of all available values was calculated).

#### Quality control

To exclude unhealthy neurons and suboptimal recording conditions, we only included neurons for cellular property (Extended Data Fig. 1) and cluster analyses when they met the following criteria:

- Resting membrane potential between −90 mV and −60 mV (without liquid junction potential correction)
- Access resistance of <50 MΩ (24 ± 10 MΩ, mean ± s.d.)
- Cortical depth of <1,200 μm

Of 1,560 whole-cell patch-clamp recordings, 1,096 neurons met these inclusion criteria. For 920 of these neurons, all 15 electrophysiological properties, as well as a value for the cortical depth, could be extracted, and they were used for clustering analyses. For most analyses, we focused exclusively on pyramidal neurons. Of the 1,560 recorded neurons, 81 were labeled as interneurons by experimenters based on cellular electrophysiological properties or inhibitory synaptic output. For 50 interneurons with successful anatomical staining, this labeling was confirmed by the neuron’s morphology (see below). Furthermore, experimenter labeling was confirmed by unsupervised clustering of 920 neurons with complete data, which objectively identified a group of 13 neurons with interneuron characteristics (see below). Excluding the 81 interneurons yielded a pyramidal neuron dataset of 1,479 neurons (Extended Data Fig. 1). For cellular property analyses of pyramidal neurons, we used 901 cells, which fulfilled the inclusion criteria listed above and had complete data (Extended Data Fig. 1). For a subset of analyses, we applied looser inclusion criteria and included pyramidal neurons with missing data values for cellular properties (*n* = 163), resting membrane voltage between −60 mV and −50 mV (*n* = 109) and cells with measured access resistance of >50 MΩ but decent electrophysiological recordings upon visual inspection by experienced patch-clamp electrophysiologists (*n* = 41), yielding a dataset with 1,214 pyramidal neurons (Extended Data Fig. 1).

### Synaptic physiology analysis

#### Detection of monosynaptic connections

As described previously^30^, we probed for monosynaptic connections for up to ten simultaneously recorded neurons. In a subset of experiments, we also used the ‘clean2extend’ approach to reuse pipettes that were cleaned in ‘Alconox’ solution to subsequently patch additional neurons^30,70^. During the connectivity recording protocol, all neurons were held close to −60 mV using the automated current injection mode of the MultiClamp Commander. To screen for synaptic connections, we elicited four APs in each neuron at 20 Hz with stimulation pulses of 1–4 nA for 1–3 ms in current clamp mode. The minimal stimulation amplitude and duration needed to elicit an AP were determined before connectivity screening. All neurons were stimulated subsequently with an interstimulus interval of 1.5 s. That means that, depending on the recording setup, the same neuron was stimulated every 12 or 15 s. A monosynaptic connection was identified when correlated postsynaptic potentials could be detected in averaged current clamp traces from 20 to 50 sweeps in any of the other simultaneously recorded neurons. Postsynaptic neurons that did not show a correlated response to presynaptic stimulation were classified as unconnected. All identified monosynaptic connections underwent an additional quality control and data curation (see below).

#### Properties of EPSPs

We developed custom MATLAB scripts to automatically extract features of the EPSPs from the raw files. We first computed the EPSP baseline (10-ms interval before presynaptic AP stimulus), EPSP amplitude (peak of moving mean of 0.5-ms sliding window in interval of 0–20 ms after maximum upstroke time of presynaptic AP) and the amplitude and peak time of the presynaptic AP in single sweep current clamp traces. We used these sweepwise parameters to automatically exclude sweeps with bad recording quality based on the following exclusion criteria: postsynaptic baseline more positive than −45 mV or more negative than −80 mV, EPSP amplitude above 20 mV (excludes postsynaptic APs), presynaptic AP amplitude below −50 mV (potentially not triggered AP) and presynaptic AP peak time within 0.5 ms of stimulus (avoids stimulus artifact).

After automated sweep exclusion, we computed synaptic parameters of each identified connection based on averaged EPSP traces of the included sweeps (in most cases, 30–50 sweeps). The EPSP baseline was calculated based on the average of 10 ms before AP stimulus. EPSP amplitude was determined by the peak of a moving mean (0.5-ms sliding window) in an interval between 0.7 ms (to avoid stimulus artifact) to 20 ms after the maximum slope time of presynaptic AP relative to the baseline. The same procedure was used to compute the amplitude of the second EPSP that is elicited by the second presynaptic AP, with the EPSP baseline computed before the second presynaptic AP stimulus. The PPR was calculated as the ratio of the second-to-first EPSP. The EPSP latency was determined as the time between maximum slope time of the presynaptic AP and the EPSP onset time. The EPSP onset was automatically determined by a series of steps to—(1) find negative peak of postsynaptic trace of 0.7 ms after stimulation (avoid stimulation artifact) and before EPSP peak as start of detection interval, (2) use maximum slope time of EPSP as end of detection interval, (3) calculate a moving mean (0.5-ms sliding window) of the derivative of the averaged postsynaptic EPSP (that is, smoothed slope) and (4) identify EPSP onset as the time point when the smoothed slope exceeded thrice of the standard deviation during baseline (calculated on derivative in 30-ms interval before next AP). These steps enabled the reliable detection of the EPSP onset and thereby the synaptic latency, which was verified through the manual curation process (see below). Please note that synaptic latencies below 0.7 ms could not be discerned due to overlap with stimulation artifacts and were assigned the value of 0.7 ms. The EPSP maximum slope was calculated within the interval between the negative peak (see step 1 above) and the EPSP peak time. The EPSP halfwidth was calculated as the time between the EPSP trace crossing above and below 50% of its peak amplitude. Notably, it was only computed in those connections where the EPSP decayed to below 50% of its peak value within the 50-ms interstimulus interval.

#### Curation of synaptic dataset

To verify the automatic parameter extraction and to ensure a high quality of our synaptic dataset, we curated every single neuron pair that was classified as being connected during the initial connectivity screening as part of the recordings. Synapse curation was performed by visual inspection of plots generated to display both the raw and averaged traces and the automatically extracted parameters.

This was accomplished through visual inspection of traces, as well as extracted parameters, followed by manual curation as necessary. In detail, for each synapse, an overview plot was generated displaying presynaptic and postsynaptic traces included and excluded from analysis, as well as the average trace with all extracted parameters (EPSP baseline, peak, latency and halfwidth). In the first step, each connection identified during experiments was labeled as ‘certain’ or ‘uncertain’ based on these raw traces and averages. All synapses were then further assigned to subgroups based on response properties and the correctness of parameter extraction. This included synapses for which only peak and latency were deemed suitable for further analysis, faulty PPR extraction, multiple peak responses, putative EPSP–IPSP sequence or possible polysynaptic responses. Such flagged recordings were then manually curated, including visual assessment of the original recordings, decision about connection or sweep exclusion or about omission or replacement of specific parameters by manually measured values. Visual assessment and data curation of traces were done by five highly experienced patch-clamp electrophysiologists (H.P., F.X.M., H.A, J.R.P.G. and Y.P.), with group assignment and manual adjustment decisions for individual measurements taken by one of the researchers and verified by the rest of the group. During this process, of all initially classified 1,466 monosynaptic connections, 450 connections were flagged and underwent manual data curation (the following categories are not mutually exclusive)—47 connections were determined as false positives and relabeled as ‘not connected’, because of the absence of correlated EPSP. We curated the synaptic amplitude of 245 connections—53 connections did not have sufficient recording quality and we removed the amplitude value (for example, due to depolarized postsynaptic membrane potential or too few sweeps), and 92 connections required either manual sweep selection or remeasurement using the respective commercial recording software. We curated the synaptic latency of 94 connections—65 connections did not have sufficient recording quality and we removed the latency value, and 29 connections required manual remeasurement. We curated the synaptic halfwidth of 197 connections—all of these did not have sufficient recording quality to reliably detect halfwidth and we removed the halfwidth value; thus, no manual remeasurement was attempted for this parameter. A total of 46 connections were identified as exhibiting disynaptic or polysynaptic interference on top of the monosynaptic response and were thus only used for the connection probability analysis (Extended Data Fig. 1). The curation decisions and remeasurements were documented in a table and automatically applied to the dataset through custom-written MATLAB scripts. For different analyses of the synaptic dataset, these criteria were specifically combined with cellular and morphological inclusion criteria to ensure strict quality control of each analysis (Methods; Extended Data Fig. 1). As a result of the strict quality control and extensive curation procedure, synaptic measurements of the averaged traces were sufficiently reliable, and no model fitting procedure was needed.

### Morphological analysis

#### Cell classification

Of the 203 recorded clusters, successful anatomical staining was achieved in 169 clusters, encompassing a total of 1,162 neurons that could be assigned to the electrophysiology dataset (75% of all recorded neurons). These neurons were classified into 1,112 pyramidal neurons by their characteristic morphology (a vertical somatodendritic domain with a prominent apical dendrite running toward the pia mater and forming a tuft in L1, and basal dendrites fanning out from the opposite side of the soma), as well as 50 interneurons (for example, based on the dendritic and axonal arborization pattern). The morphologically identified interneurons were excluded from the dataset.

#### Alignment of cells and their cortical positions

During the patch-clamp recordings, relative soma positions were documented using the software of the DIC infrared microscope (Olympus cellSens). These soma location coordinates in the acute slices were then used to align and scale the soma coordinates obtained from the immunohistochemical staining using a custom-written MATLAB script. The distance from the pia vertically to the soma of each neuron was taken as their cortical depth. From 85 neurons, no coordinates were obtained during recording; these neurons and their synapses were excluded from depth-based analyses (Extended Data Fig. 1).

#### Morphometric feature analysis

A total of 70 pyramidal neurons were selected for morphological feature extraction according to the following procedure: in the first step, clusters with intact morphology (including apical and basal dendrites) and intact axons were chosen, and, in the second step, at least 10–20 neurons of each e-type were selected. Reconstructed neurons were analyzed using custom-written ‘hoc’ scripts^71^ in the NEURON software environment^72^. A Gaussian spatial filter was applied to the reconstructed vectorial structure (three-point window, three iterations in the *xy* plane and five iterations for values along the *z* axis). *Z* shrinkage was compensated by using a correction factor applied to the *z* axis of the reconstructed neuron. Sholl analysis was performed using the SNT plugin of the Fiji/ImageJ software package^73^.

#### Detection of slice cutting artifacts for connectivity analysis and assessment of axon truncation effects on electrophysiology

The slicing procedure of acute brain tissue is necessary for high-resolution patch-clamp recordings. During this process, special care is taken to maintain an optimal cutting angle to avoid cutting dendrites and axons, as this can lead to an underestimation of synaptic connection probability. We optimized the slicing procedure to only cut parallel to the apical dendritic axis (perpendicular to the cortical surface). To confirm the parallel cut and the preservation of axons after biocytin staining, we estimated the cutting angle of the slices based on the orientation of the apical dendrites relative to the slice surface and assessed the axon of each recorded and stained pyramidal neuron (*n* = 1,112). A total of 25 slices exhibited dendrites that were not parallel to the slice surface, with the basal domain and axon pointing toward the slice surface. These slices included 166 pyramidal neurons that were excluded from the connection probability analysis (Extended Data Fig. 1a). In the remaining slices, 84 pyramidal neurons had an axon length of less than 100 µm and were also excluded from the connection probability analysis if not having a previous collateral (Extended Data Fig. 1a). Axon truncation information was also used to assess and exclude possible effects of early axon truncation on intrinsic properties and thus e-type identity of the neurons (Extended Data Fig. 10)^74^.

### Statistics and reproducibility

Tissue from all patients admitted to the hospital for resective temporal pole epilepsy surgery due to drug-resistant epilepsy was generally included, with the prerequisite that prior written informed consent for the scientific use of resected tissue was obtained. There was no systematic recruitment according to sex, age or other variables. The main goal of this study was to describe diversity of cellular and synaptic properties in a large dataset. No statistical method was therefore used to predetermine sample sizes. Only data with large experimental yield from single patients (25 or more pyramidal cells) were included, and neurons with a distance of more than 1,200 µm from the pia were excluded from analyses. Cellular and synaptic property analyses were performed on curated datasets to ensure quality of the electrophysiological data (‘Quality control’ and ‘Curation of synaptic dataset’; Extended Data Fig. 1). Final sample sizes were a result of data curation procedures as well as e-type classification and not predetermined. No experimental groups were defined in this study. Data collection by three experimenters (F.X.M., Y.P. and H.P.) was therefore not randomized and the investigators were not blinded to allocation during experiments and to outcome assessment.

### Statistical and cluster analyses

Statistical analyses and data visualization were performed using R^75^ and the IDE RStudio^76^. Unless otherwise specified, the ‘tidyverse’ R package^77^ combined with Affinity Publisher (Serif Europe), CorelDRAW (Corel) or Inkscape (Software Freedom Conservancy) was used to visualize the data. The R packages used for statistical analyses are cited in the respective sections below. Distributions of intrinsic and synaptic data could often not be assumed to be normal (Fig. 2 and Extended Data Fig. 3) and statistical tests not assuming normal distribution and homogeneity of variances were chosen. In some cases (Extended Data Figs. 5 and 10), normal distribution and equality of variances were not formally tested. Individual data points were only excluded from further analyses and, therefore, from statistical testing during the data curation procedure, as described above.

#### Linear regression models, linear mixed-effects models and distribution models

The ‘lm’ function from the ‘stats’ package^75^ was used to fit linear regression models. To test for statistical significance, regression models were compared to intercept-only models using an *F* test. Linear mixed-effects models were fitted to the data using the ‘lme4’ package^78^. For analysis of cortical depth dependence of electrophysiological cellular properties among participants, we specified cortical depth as a fixed effect and modeled random intercepts and slopes by human participants—‘electrophysiological property ~ cortical depth + (1 + cortical depth | human participant)’. The ‘fitdist’ function from the ‘fitdistrplus’ package^79^ was used to fit Gaussian and log-normal distributions to the empirical EPSP amplitude distributions of individual human participants (Extended Data Fig. 2b). For each participant, the resulting Gaussian and log-normal models were then compared using the Akaike information criterion.

#### Correlation analysis and PCA of cellular properties

The correlation matrix containing Spearman rank correlation coefficients of the 15 electrophysiological properties was calculated using the ‘stats’ package and visualized using the ‘ggcorrplot’ function. The PCA of the 15 electrophysiological properties was performed using the ‘prcomp’ function of the ‘stats’ package. Before performing PCA, the data were standardized (*z* scores).

#### Unsupervised hierarchical clustering analysis

We performed an unsupervised hierarchical cluster analysis based on the 15 electrophysiological properties^80,81^. We included all neurons that passed quality control and had complete data (*n* = 920) and used the ‘hclust’ function of the ‘stats’ package. Before clustering, the data were standardized (*z* scores). Euclidean distance across data points was calculated and ‘ward.D2’ linkage criterion was used as the agglomeration method^82^. The resulting dendrogram was cut at the level of two groups. One of the two resulting groups contained 13 neurons with clear fast-spiking interneuron characteristics, as determined by manual inspection. The other 907 neurons, which had properties typical for pyramidal neurons, were clustered again using unsupervised hierarchical clustering (Fig. 3c; Euclidean distance and ‘ward.D2’ method). The ‘sigclust2’ package was used to test the statistical significance of the hierarchical clustering using a Monte Carlo-based approach^63^. This method sequentially tests whether the data at each split correspond to one (*H*_0_) or more multivariate Gaussian distributions (*H*_1_) while controlling for the family-wise error rate (significance level was set to *α* of 0.05). To screen for an appropriate number of clusters, we used the ‘NbClust’ function from the ‘NbClust’ package^83^ to automatically calculate 26 different indices for determining the number of clusters. The results of these different indices suggested that two, three or four clusters would be most appropriate given our dataset. We further used a resampling method to narrow down the appropriate number of clusters and to address how the clustering result is affected by changes in the dataset (Extended Data Fig. 4)^84^. The approach can be outlined as follows: step 1—perform unsupervised hierarchical clustering of the pyramidal neuron dataset into *k* = *n* clusters. Step 2—draw 80% random sample and perform hierarchical clustering of the subsample into *k* = *n* clusters. Match the clusters obtained in steps 1 and 2 by pairing the medoids based on their Euclidean distance. Step 3—calculate the fraction of cells in the subsample that were correctly classified into corresponding clusters in steps 1 and 2. Repeat steps 2 and 3 for 10,000 rounds (Extended Data Fig. 4d,g). This resampling approach was performed for *k* = 3, *k* = 4 and *k* = 5 clusters. We obtained the highest median fraction of correctly reclassified pyramidal neurons when the data were clustered into *k* = 4 clusters (Extended Data Fig. 4g). Taken together, we propose that the cellular parameter space analyzed in this study can be best described with four functional groups of pyramidal neurons. To find the most distinguishing cellular properties for pyramidal neuron clusters, we compared each cluster against all other pyramidal neurons for each cellular property and selected the properties yielding the highest *F* values (‘aov’ function from the ‘stats’ package). To validate whether other clustering algorithms yield similar results, we additionally performed *k*-means clustering (‘stats’ package, Hartigan–Wong method^85^) of the pyramidal neuron dataset into *k* = 4 clusters. We used randomly selected starting points (*n* = 10,000) and allowed 10,000 iterations. The *k*-means clustering yielded a very similar result compared to hierarchical clustering (Extended Data Fig. 4h). In addition, 6 of the 907 neurons that were clustered into the pyramidal neuron group in the initial clustering step were labeled as potential interneurons by experimenters and, as a conservative measure, were manually removed after clustering analyses, yielding a pyramidal neuron dataset of 901 neurons (Extended Data Fig. 1). Pyramidal neurons with missing data values for cellular properties (*n* = 163), resting membrane voltage between −60 mV and −50 mV (*n* = 109) and cells with measured access resistance of >50 MΩ but decent electrophysiological recordings upon visual inspection by experienced patch-clamp electrophysiologists (*n* = 41), were recovered and classified for a subset of analyses (Extended Data Fig. 1). For these neurons, missing data values were aggregated using the ‘na.aggregate’ function from the ‘zoo’ package^86^. This function replaces missing values by the mean of all available values for a certain parameter. The recovered neurons were then assigned to e-type groups using *k*-nearest neighbor algorithm from the ‘class’ package^87^ with the hierarchical clustering of the strictly curated dataset acting as the training dataset. For combined morphoelectric data analysis (Extended Data Fig. 7), a PCA with a subsequent hierarchical clustering (Wards method) was performed using MATLAB (2022b; The MathWorks).

#### Correspondence analysis

We performed a correspondence analysis between our e-types and t-types, which were previously published for L2–3 in ref. ^10^. The eight parameters AP threshold, AP height, AP width at half height, max upstroke, max downstroke, input resistance, resting membrane potential, as well as sag ratio^10^ were deemed to be well-comparable to AP threshold, AP Amplitude, AP halfwidth, AP upstroke, AP downstroke, input resistance, resting membrane potential, as well as sag ratio measures of our study, respectively. These parameters were included in correspondence analyses, including conversion to ms and inversion of signs in some cases and with −1/sag ratio (ref. ^10^) + 1 = sag ratio of our study. The remaining parameters were extracted according to slightly different protocols or were only reported in one of the studies and were therefore omitted. A class-balanced logistic regression classifier was constructed^10^ using the selected variables. It performed similarly to the one presented in the original study (‘Results’; Extended Data Fig. 6a). In a second step, the classifier was used to assign pseudo-t-type labels to neurons in our dataset based on their electrophysiological data (Extended Data Fig. 6b,c).

#### UMAP dimensionality reduction

To visualize our 15-dimensional parameter space (15 electrophysiological properties), we used the uniform manifold approximation and projection (UMAP) technique for dimensionality reduction (‘umap’ package^88,89^). The data were standardized (*z* score) before performing dimensionality reduction. The UMAP figures were plotted using the following hyperparameters: n_neighbors = 100, spread = 0.05, min_dist = 5 × 10^−5^. To generate patient-specific UMAP plots, the coordinates of the UMAP projection of the pooled data were used, but only patient-specific data points were visualized.

### Reporting summary

Further information on research design is available in the Nature Portfolio Reporting Summary linked to this article.

## Online content

Any methods, additional references, Nature Portfolio reporting summaries, source data, extended data, supplementary information, acknowledgements, peer review information; details of author contributions and competing interests; and statements of data and code availability are available at 10.1038/s41593-025-02134-7.

## Supplementary information

Reporting Summary

## Data Availability

The processed data and the code for analysis and visualization are available online via figshare (10.6084/m9.figshare.28184126)^90^. Original data from human participants are not shared in a public open-access fashion due to EU regulations for data protection (GDPR) and their implementation to German law.

## Code availability

The code for analysis and visualization is available online via figshare (10.6084/m9.figshare.28184126)^90^.
