# Supplementary material for: Electrophysiological classification of human layer 2–3 pyramidal neurons reveals subtype-specific synaptic interactions
Source: Nat Neurosci. 2025 Dec 10;29(2):455–66. doi: 10.1038/s41593-025-02134-7 (PMC12880919; doi:10.1038/s41593-025-02134-7)
Supplement: Supplementary file 1 — Reporting Summary [file 41593_2025_2134_MOESM1_ESM.pdf]

nature portfolio

Corresponding author(s): Jörg R. P. Geiger, Henrike Planert, Yangfan Peng, Imre Vida

Last updated by author(s): Sep 29, 2025

Reporting Summary

Nature Portfolio wishes to improve the reproducibility of the work that we publish. This form provides structure for consistency and transparency in reporting. For further information on Nature Portfolio policies, see our Editorial Policies and the Editorial Policy Checklist.

Statistics

For all statistical analyses, confirm that the following items are present in the figure legend, table legend, main text, or Methods section.

n/a	Confirmed	
☐	☒	The exact sample size (*n*) for each experimental group/condition, given as a discrete number and unit of measurement
☐	☒	A statement on whether measurements were taken from distinct samples or whether the same sample was measured repeatedly
☐	☒	The statistical test(s) used AND whether they are one- or two-sided<br>*Only common tests should be described solely by name; describe more complex techniques in the Methods section.*
☐	☒	A description of all covariates tested
☐	☒	A description of any assumptions or corrections, such as tests of normality and adjustment for multiple comparisons
☐	☒	A full description of the statistical parameters including central tendency (e.g. means) or other basic estimates (e.g. regression coefficient) AND variation (e.g. standard deviation) or associated estimates of uncertainty (e.g. confidence intervals)
☐	☒	For null hypothesis testing, the test statistic (e.g. *F*, *t*, *r*) with confidence intervals, effect sizes, degrees of freedom and *P* value noted<br>*Give P values as exact values whenever suitable.*
☒	☐	For Bayesian analysis, information on the choice of priors and Markov chain Monte Carlo settings
☐	☒	For hierarchical and complex designs, identification of the appropriate level for tests and full reporting of outcomes
☐	☒	Estimates of effect sizes (e.g. Cohen's *d*, Pearson's *r*), indicating how they were calculated

Our web collection on statistics for biologists contains articles on many of the points above.

Software and code

Policy information about availability of computer code

Data collection	Olympus Cell Sens, Excel (Microsoft Corporation), pClamp 10 (Molecular Devices) and Signal 6 (Cambridge Electronic Design), Custom-made software used for semiautomated multipatch measurements in Matlab (Mathworks), as published previously (Peng et al. 2019).
Data analysis	Morphological reconstruction and analysis: Neutube software package (Feng et al. 2015), custom written ‘hoc’ scripts in the NEURON software environment (Degro et al. 2015), Fiji/ImageJ software package (Schindelin 2012). Electrophysiological data: a custom-written automated data analysis pipeline (Matlab, Mathworks) was used, as was for the alignment of cell coordinates from Olympus CellSens software and their laminar positions in histochemically stained slices. Further data analysis and visualization: R (R core team, Vienna); data vizualisation in combination with Corel Draw (Corel Corporation), Inkscape (Software Freedom Conservancy) and Affinity Publisher (Serif). The code for analysis and visualization is made available upon publication.

For manuscripts utilizing custom algorithms or software that are central to the research but not yet described in published literature, software must be made available to editors and reviewers. We strongly encourage code deposition in a community repository (e.g. GitHub). See the Nature Portfolio guidelines for submitting code & software for further information.

Data

Policy information about availability of data

All manuscripts must include a data availability statement. This statement should provide the following information, where applicable:

- Accession codes, unique identifiers, or web links for publicly available datasets
- A description of any restrictions on data availability
- For clinical datasets or third party data, please ensure that the statement adheres to our policy

The processed data and the code for analysis and visualization is available online (10.6084/m9.figshare.28184126). Original data from human subjects is not shared in a public open-access fashion due to EU regulations for data protection (GDPR) and their implementation to German law.

Research involving human participants, their data, or biological material

Policy information about studies with human participants or human data. See also policy information about sex, gender (identity/presentation), and sexual orientation and race, ethnicity and racism.

Reporting on sex and gender	Data from both sexes was analysed in this study. The data obtained was measured in acute brain slices obtained from temporal lobe resections in 23 patients suffering from drug-resistant epilepsy (12 male, 11 female).
Reporting on race, ethnicity, or other socially relevant groupings	No socially relevant grouping variables are used in this study.
Population characteristics	All patients had drug-resistant epilepsy. The age range was 21 to 55 years with a median age of 34 years.
Recruitment	Tissue from all patients admitted to the hospital for resective temporal pole epilepsy surgery due to drug-resistant epilepsy was generally included, with the prerequisite that prior written informed consent for the scientific use of resected tissue was obtained. There was no systematic recruitment according to sex, age or other variables.
Ethics oversight	The study procedures adhered to all relevant ethical regulations and were approved by the local ethical committee (Ethikkommission der Charité - Universitätsmedizin Berlin) with approval number EA2/111/14.

Note that full information on the approval of the study protocol must also be provided in the manuscript.

Field-specific reporting

Please select the one below that is the best fit for your research. If you are not sure, read the appropriate sections before making your selection.

☒ Life sciences ☐ Behavioural & social sciences ☐ Ecological, evolutionary & environmental sciences

For a reference copy of the document with all sections, see nature.com/documents/nr-reporting-summary-flat.pdf

Life sciences study design

All studies must disclose on these points even when the disclosure is negative.

Sample size	In this study due to high-throughput sampling more datapoints (more than 1400 cells and 1400 synapses in the entire pyramidal neuron dataset) than in previous studies could be assessed, making it possible to investigate general cellular principles of L2-3 pyramidal neurons and their synapses. Only data with large experimental yield from single patients (25 or more cells) were included to enable analyses on the individual patient level.
Data exclusions	Less than 25 pyramidal cells per patient, cells with more than 1200 µm distance to pia.
Replication	Reproducibility of cellular and synaptic principles was tested at the single-patient level. Generally attempts at reproducing cellular and synaptic principles within or across individuals were successful unless otherwise specified in the results section of the manuscript.
Randomization	No experimental groups were defined in this study.
Blinding	Blinding is not relevant as no experimental groups were defined.

Reporting for specific materials, systems and methods

We require information from authors about some types of materials, experimental systems and methods used in many studies. Here, indicate whether each material, system or method listed is relevant to your study. If you are not sure if a list item applies to your research, read the appropriate section before selecting a response.

Materials & experimental systems

n/a	Involved in the study
☒	☐ Antibodies
☒	☐ Eukaryotic cell lines
☒	☐ Palaeontology and archaeology
☒	☐ Animals and other organisms
☒	☐ Clinical data
☒	☐ Dual use research of concern
☒	☐ Plants

Methods

n/a	Involved in the study
☒	☐ ChIP-seq
☒	☐ Flow cytometry
☒	☐ MRI-based neuroimaging

Plants

Seed stocks

Report on the source of all seed stocks or other plant material used. If applicable, state the seed stock centre and catalogue number. If plant specimens were collected from the field, describe the collection location, date and sampling procedures.

Novel plant genotypes

Describe the methods by which all novel plant genotypes were produced. This includes those generated by transgenic approaches, gene editing, chemical/radiation-based mutagenesis and hybridization. For transgenic lines, describe the transformation method, the number of independent lines analyzed and the generation upon which experiments were performed. For gene-edited lines, describe the editor used, the endogenous sequence targeted for editing, the targeting guide RNA sequence (if applicable) and how the editor was applied.

Authentication

Describe any authentication procedures for each seed stock used or novel genotype generated. Describe any experiments used to assess the effect of a mutation and, where applicable, how potential secondary effects (e.g. second site T-DNA insertions, mosiacism, off-target gene editing) were examined.
